# Higher evolutionary dynamics of gene copy number for Drosophila glue genes located near short repeat sequences

**DOI:** 10.1186/s12862-023-02178-y

**Published:** 2024-02-02

**Authors:** Manon Monier, Isabelle Nuez, Flora Borne, Virginie Courtier-Orgogozo

**Affiliations:** 1 Université Paris Cité, CNRS, Institut Jacques Monod, 75013 Paris, France; 2 Department of Biological Sciences, Columbia University, New York city, New York, USA

**Keywords:** Glue genes, Bioadhesive, Sgs, Mucin, Gene family, Gene diversification, Gene turnover, Gene loss, Gene duplication, Synteny, Repeat

## Abstract

**Background:**

During evolution, genes can experience duplications, losses, inversions and gene conversions. Why certain genes are more dynamic than others is poorly understood. Here we examine how several *Sgs* genes encoding glue proteins, which make up a bioadhesive that sticks the animal during metamorphosis, have evolved in Drosophila species.

**Results:**

We examined high-quality genome assemblies of 24 Drosophila species to study the evolutionary dynamics of four glue genes that are present in *D. melanogaster* and are part of the same gene family - *Sgs1, Sgs3, Sgs7 and Sgs8 -* across approximately 30 millions of years. We annotated a total of 102 *Sgs* genes and grouped them into 4 subfamilies. We present here a new nomenclature for these *Sgs* genes based on protein sequence conservation, genomic location and presence/absence of internal repeats. Two types of glue genes were uncovered. The first category (*Sgs1, Sgs3x, Sgs3e*) showed a few gene losses but no duplication, no local inversion and no gene conversion. The second group (*Sgs3b, Sgs7, Sgs8*) exhibited multiple events of gene losses, gene duplications, local inversions and gene conversions. Our data suggest that the presence of short “new glue” genes near the genes of the latter group may have accelerated their dynamics.

**Conclusions:**

Our comparative analysis suggests that the evolutionary dynamics of glue genes is influenced by genomic context. Our molecular, phylogenetic and comparative analysis of the four glue genes *Sgs1, Sgs3, Sgs7* and *Sgs8* provides the foundation for investigating the role of the various glue genes during Drosophila life.

**Supplementary Information:**

The online version contains supplementary material available at 10.1186/s12862-023-02178-y.

## Background

Genes can be grouped into gene families when they share a common ancestor and are present either in distinct genomes (orthologs and paralogs) or within a single genome (paralogs) due to gene duplications [1]. The increase in gene copy number in a genome can have several fitness advantages: to increase the amount of products (e.g., ribosomal RNAs), to diversify protein activity (e.g., opsins) and to diversify gene expression patterns (e.g., Hox transcription factors) [2]. Gene duplications and gene losses are frequently involved in phenotypic evolution and adaptation [2–5]. In humans, on a per nucleotide basis, gene copy number differences between individuals represent an even larger pool of genetic variation available to selection than single nucleotide polymorphisms [1, 6].

Certain genes are found to exhibit accelerated rates of gene turnover and several factors have been proposed to explain why the pace of gene duplication and gene loss can differ between genes. A first type of explanation relates to the selective forces that act on genes. For example, genes involved in interactions with the environment such as chemoreception, reproduction, stress response or immune defense are generally expected to adapt faster due to conditions that change more rapidly and indeed they are usually observed to undergo faster gene turnover than average genes [1, 7]. In contrast, a few particular genes may require strict stoichiometric balance due to their interactions with other proteins and are less likely to vary in gene copy number [8–10]. A second type of explanation considers the rate of the mutation process itself. Structural changes and thus gene turnover can be facilitated by the presence of certain elements in the genome, such as repeated sequences [11], transposable elements [12] or fragile DNA regions that are more susceptible to DNA breakage [13].

Duplicated gene copies are often clustered at specific genomic locations [14]. Examining the immediate surroundings of gene copies, researchers have often noticed the presence of transposable elements, for example for pigmentation transcription factor genes in maize [15], effector genes in grass powdery mildew [16], insecticide resistance genes in Drosophila [17], amylase genes in Vertebrates [18] and fatty acid metabolic genes in fish [19]. Transposable elements usually flank genes and are oriented in the same direction. They provide regions of high sequence identity that can be used as templates for unequal crossing overs, resulting in the removal or duplication of gene coding sequences between the two elements [12].

The increasing number of available full genome sequences from a variety of organisms offers an unprecedented opportunity to investigate more thoroughly the tempo of gene turnover and the evolutionary forces controlling gene gains and losses. High quality assemblies are required to correctly infer the rates of gene turnover. In case of sequencing errors, certain gene copies and short open-reading frames can be missed. Errors in genome assemblies can also lead to the fragmentation of genes into several individual contigs, the withdrawal of recent duplicates, the split of heterozygous single-copy genes or even sometimes the incorporation of gene sequences from contaminant species [20]. Such incorrect assessment of the number of gene copies within genomes usually lead to higher estimates of the rates of gene gains and losses [21]. On the other hand, comparing species that are too distantly related can overlook rapid duplications followed by the elimination of one of the extra gene copy and lead to an underestimation of gene turnover rates. Overall, gene turnover is best assessed with closely related species and genomes based on long-read sequencing methods. To help in finding ortholog genes and confirming potential gene losses, it can also be useful to perform whole-genome alignments, determine syntenic regions where genes are expected to occur and then search for the presence of the genes of interest in the syntenic region [22].

The Drosophila glue genes, also named Salivary gland secretion (*Sgs*) genes, represent a simple and attractive model system to study the evolutionary forces acting on the evolutionary dynamics of gene copies [23]. These genes encode secreted proteins that make up a bioadhesive that allows the animal to attach itself to a surface for several days while it remains still during metamorphosis [24]. The glue of diverse Drosophila species is thought to evolve rapidly to stick to various substrates in diverse environmental conditions [24]. The specificity of Drosophila glue genes, with the exception of *Eig71Ee* (see below), is that they have only one known function, glue production. Compared to genes with multiple functions, they are thus presumably subjected to more defined and precise selective forces, which might facilitate our understanding of their evolutionary dynamics. In addition, assessing the diversity of glue genes encoded by different Drosophila species may help to identify key components of Drosophila glue adhesiveness and develop new bioadhesives.

In *Drosophila melanogaster* eight glue genes have been identified [24]. Five of them, − *Sgs1 (2 L:25B4), Sgs3 (3 L:68C11), Sgs7* (3 L:68C11), *Sgs8* (3 L:68C11) and *Eig71Ee* (3 L:71E5) harbor a phase 1 intron at the same position, which interrupts the signal peptide, and are considered to be part of the same gene family [25], The three other genes - *Sgs4* (X:3C11–12), *Sgs5* (3R:90B3–5) and *Sgs5bis* (3R:90B3–5) - have no intron (for *Sgs4*) or harbor two introns at other positions (for *Sgs5* and *Sgs5bis*). Their relationships with respect to the other glue genes have not been characterized. *Sgs1*, *Sgs3, Sgs4* and *Eig71Ee* encode for long, highly O-glycosylated proteins containing a large, disordered region harboring repeat sequences rich in proline, serine and threonine [24]. The repeat region is characteristic of mucins, which usually form a mucus which can act as a physical barrier against mechanical damage or pathogens [26]. *Sgs5, Sgs5bis, Sgs7* and *Sgs8* genes encode for shorter and more ordered proteins that are rich in cysteine and devoid of internal repeats [24]. All the *D. melanogaster* glue genes are only expressed in the salivary glands at the third instar larval stage and only known to be involved in glue production [24], with the exception of *Eig71Ee*, which is also expressed in hemocytes and in the gut, where it appears to contribute to coagulation and bacterial entrapment [27]. In a previous study [25], the rate of gene gains and losses for the *Sgs1-Sgs3-Sgs7-Sgs8* gene family was found to be significantly higher than for average genes. Here, after clarifying the relationships between the eight glue genes of *D. melanogaster,* we focus on the evolution of four glue genes: *Sgs1*, *Sgs3*, *Sgs7* and *Sgs8*. We use recently published high quality assemblies of closely related species of Drosophila flies [28] to reconstruct their evolutionary dynamics across approximately 30 million years of evolution. We observe that the rates of gene duplication, gene inversion and gene conversion vary between genes, and we explore the possible effect of genomic context on gene dynamics.

## Results

### Two families of glue genes in *D. melanogaster*

**Fig. 1:**
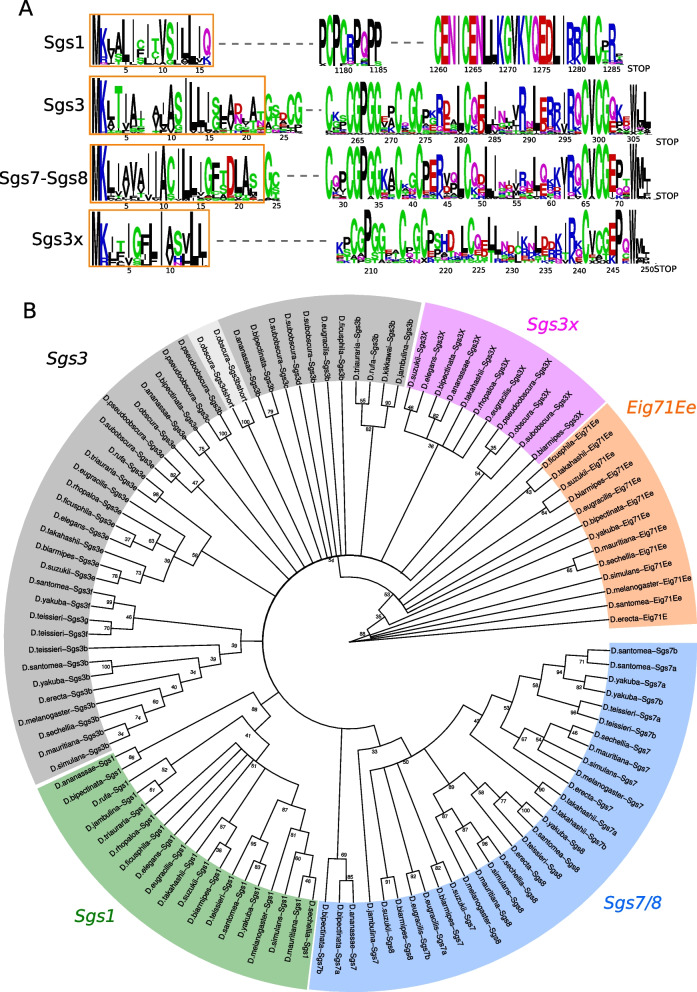
Overview of the Sgs1-Sgs3-Sgs7-Sgs8 protein family in Drosophila. **A** Conserved amino acid motifs in Sgs proteins. The column height indicates conservation of the sequence at that position while the height of the amino acids within the column shows relative frequency. Orange boxes indicate conserved sequences within signal peptides. Dotted lines indicate blocks of less conserved amino acid sequences. Numbers indicate the positions of the amino acid in the corresponding *D. melanogaster* protein, or in *D. suzukii* for Sgs3x as this protein is absent in *D. melanogaster.* All the *Sgs1–3–7-8* genes contain a phase 1 intron disrupting the signal peptide sequence whose position corresponds to amino acid position 10. **B** Maximum likelihood unrooted tree of Eig71Ee, Sgs1, Sgs3, Sgs3x, Sgs7 and Sgs8 amino acid sequences from all studied species. Gene names and colors were attributed based on synteny information (see text for details). Numbers on branches represent bootstrap values. Note that most bootstrap values are low, due in part to the small number of amino acids composing the Sgs7 and Sgs8 proteins

Alignments of the amino acid sequences encoded by the eight glue genes of *D. melanogaster* and their annotated orthologs from various Drosophila species [25] revealed that Drosophila glue genes form two distinct gene families and that there is no sequence match between them besides the signal peptide (Fig. 1, Fig. S1, Files S1–2). The first gene family comprises *Sgs1, Sgs3, Sgs7, Sgs8* and *Eig71Ee* (Fig. 1, File S2) whereas the second gene family contains *Sgs4, Sgs5* and *Sgs5bis* (Fig. S1, File S2). Genes of the first gene family are characterized by an IRXC [L/V] C motif in the encoded C-terminal domain and the presence of a phase 1 intron disrupting the signal peptide sequence whose position corresponds to amino acid position 10 (Fig. 1A). The second family proteins display a PCXXXXK motif in the C-terminal region (Fig. S1A).

In a previous study [25], we found that for the group of *Sgs1, Sgs3, Sgs7* and *Sgs8* genes, the rate of gene gains and losses was significantly higher than for average genes. In order to examine further the evolutionary dynamics of gene copies for this glue gene family and the factors influencing their rate of evolution, we decided to take advantage of high quality genome assemblies that became available in 2021 [28]. We chose to focus on closely related species of Drosophila which diverged relatively recently, so that we were unlikely to interpret as gene copy stasis situations that resulted from rapid duplications followed by the elimination of one of the duplicated copies. In the present study, we did not analyze *Eig71Ee,* as it has a supplementary role in immune defense and is thus probably subjected to additional functional constraints compared to the other glue genes. Overall, we examined the evolutionary dynamics of four glue genes - *Sgs1, Sgs3, Sgs7* and *Sgs8 -* across 25 Drosophila species.

### Existing genome annotations are often incomplete for *Sgs* genes

Using BLAST [29], we identified and annotated all copies of the *Sgs* genes which are orthologs of *Sgs1, Sgs3, Sgs7* and *Sgs8* in high-quality genome reference sequences of *D. melanogaster* and 23 other Drosophila species (Table S1–3, File S1). Compared to previous studies of *Sgs* genes in diverse Drosophila species [25, 30], we analyzed here the genome sequence of 6 additional Drosophila species: *D. teissieri*, *D. triauraria*, *D. rufa*, *D. jambulina*, *D. obscura* and *D. subobscura*. Compared to Da Lage et al. previous study [25], which used only protein sequences from *D. melanogaster* as queries for BLAST searches, we used *Sgs* sequences from all species as BLAST queries and compared large genomic syntenic blocks between species. We thus identified 13 additional *Sgs* genes in the species examined by Da Lage et al. and annotated 13 new *Sgs* genes in genome sequences from four other species (Table S3). Furthermore, we corrected gene annotations for five *Sgs* genes in five species, where introns were absent or mislabeled (Table S3, File S1).

Da Lage et al. [25] annotated four *Sgs7* genes in *D. suzukii* based on a low-quality genome assembly [31]. Using a more recent Pacbio assembled genome [32] of the same strain, we found only one copy of *Sgs7*, located at the same position as in its closely related species *D. biarmipes*. This illustrates that determination of the number of gene copies is highly dependent on high quality genomes [20, 21]. In the present study we relied on PacBio- and Nanopore-based genome assemblies for all species, except for *D. eugracilis* and *D. takahashii* which had only Illumina-based genome sequences (Table S1).

### A new nomenclature for *Sgs3* genes

While *D. melanogaster* harbors a single *Sgs3* gene, multiple copies of this gene were previously found in several Drosophila species and were distinguished with letters *a, b, c* according to the number of copies per species and to the order of their discovery in each species [25]. Here, as we found even more *Sgs3* copies, we decided to change the gene nomenclature for better comparison between species. We define *Sgs3x* as the *Sgs3* ortholog that is deleted in the melanogaster subgroup and that is flanked in other species by the *Parg* (*CG2864*) and *Mnt* (CG13316) genes in a large genomic syntenic block, which corresponds to position 3E2 on the X chromosome in *D. melanogaster*. All the other *Sgs3* copies are in a large genomic syntenic block corresponding to region 68C10–11 on chromosome 3L *in D. melanogaster*. We labeled them from ‘b’ to ‘g’ from 5′ (near the *Chrb* gene) to 3′ (near the *CG33489* gene) according to their respective positions within this genomic locus. We note that for serendipitous reasons there is no *Sgs3a* gene in this new nomenclature. *Sgs3* genes located at the same corresponding position in the genome of diverse species were labeled with the same letter.

### Several *Sgs* genes incorrectly contained premature stop codons

The coding regions of *Sgs1* and *Sgs3* contain long internal repeats encoding motifs rich in proline, serine and threonine [24]. Premature stop codons were found in genome sequence assemblies within the repeated region of *Sgs1* in four species (*D. takahashii*, *D. rhopaloa*, *D. triauraria* and *D. ficusphila*) and of *Sgs3x* in *D. biarmipes.* Using a *D. takahashii* strain different from the genome sequence line, we PCR-amplified the region containing the presumptive premature stop codon and found an extra A nucleotide compared to the reference sequence of *Sgs1*, making up a stretch of 8 adenines instead of 7. The addition of this adenine removed the premature stop codon and gave a full length *Sgs1* coding region. In *D. triauraria* we found 6 premature stop codons dispersed throughout the 4212-bp repeated region of *Sgs1,* with frameshifts adjacent to each stop codon. The presence of repeats prevented us from amplifying the region by PCR, so we do not know whether these are genuine stop codons or sequence assembly artifacts. Analysis of raw reads from full genome sequencing projects suggests that *D. rhopaloa* *Sgs1* reference sequence may be corrected by adding an extra ‘A’ (supported by 21 reads compared to 42 reads harboring a deletion), that *D. ficusphila Sgs1* reference sequence should be corrected by removing a ‘C’ from a 6-bp stretch of C (supported by 45 reads harboring a deletion versus 10 reads an extra C) and that *D. biarmipes Sgs3x* reference sequence should be corrected by adding an extra ‘C’ (supported by 13 reads compared to 4 reads harboring a deletion) (Fig. S2, File S3). We therefore considered the modified sequences for these three species in our subsequent analysis.

In summary, we detected premature stop codons in five *Sgs* genes. Four of them likely correspond to sequence assembly errors. For *D. triauraria Sgs1*, it is not clear whether the 6 premature stop codons are real or artifactual.

### The *Sgs1, Sgs3, Sgs7* and *Sgs8* genes form four subfamilies

**Fig. 2:**
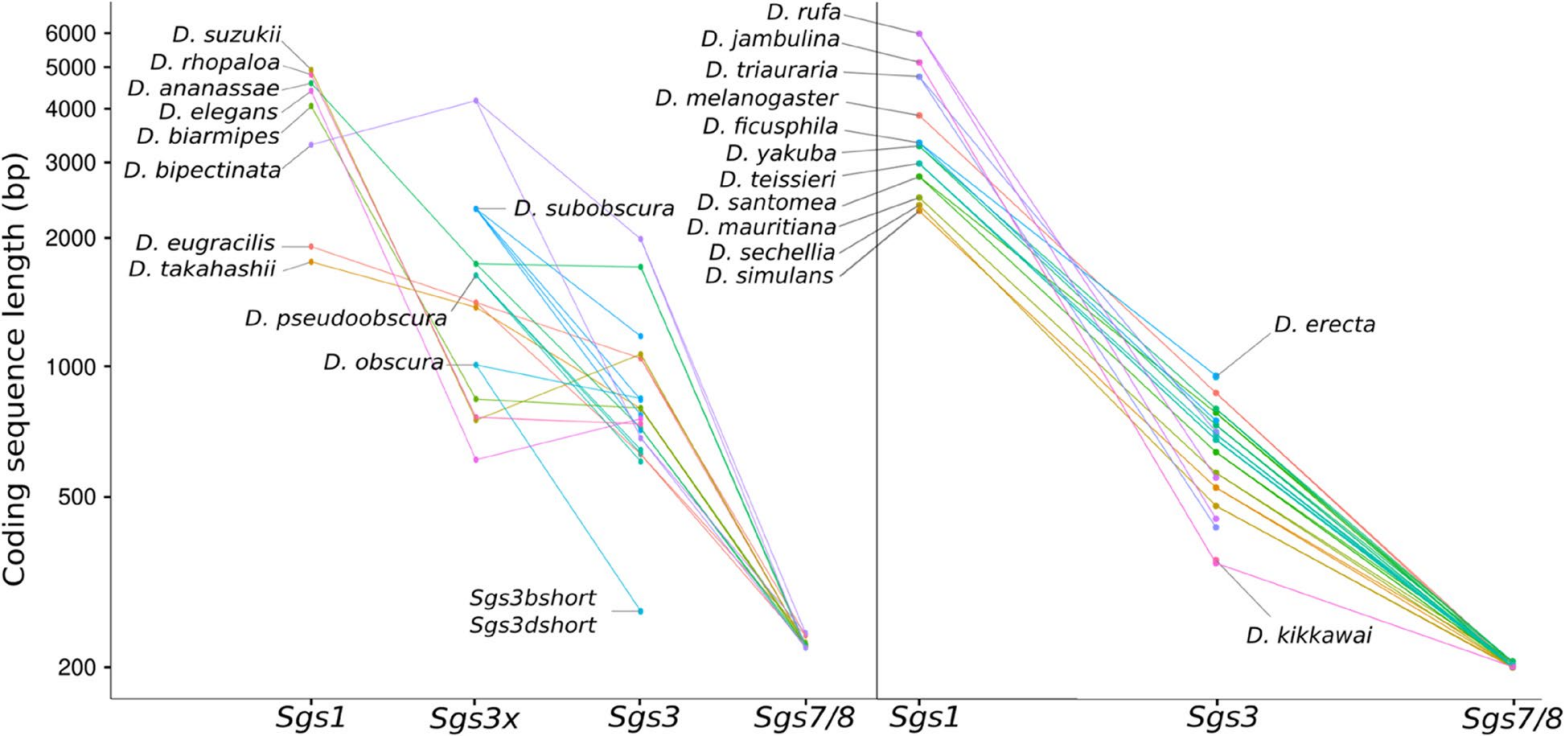
Length of *Sgs* coding sequences (with introns excluded). The y-axis is in log10 scale. Left: species which possess an *Sgs3x* gene. Right: species devoid of *Sgs3x* genes. All the 24 Drosophila species analyzed in this study are shown. For *Sgs1* in *D. triauraria*, *D. rhopaloa, D. ficusphila* and *D. takahashii* and *Sgs3x* in *D. biarmipes*, the length of the coding region was calculated as if the premature stop codons were artifacts

The four genes *Sgs1, Sgs3, Sgs7* and *Sgs8* encode proteins with a signal peptide and conserved amino acid motif patterns in the N-terminal and C-terminal regions (Fig. 1A, File S4–5). They harbor two coding exons and a short phase 1 intron interrupting the signal peptide. They can be grouped into four subfamilies based on their genomic location and synteny: *Sgs1*, *Sgs3* (which includes *Sgs3b-g* genes but not *Sgs3x)*, *Sgs3x* and *Sgs7–8* (see below for a description of each subfamily). *Sgs* coding sequence length varies greatly between genes and species, with *Sgs1* being the longest gene (higher than 1,7 kb in all species) and *Sgs7–8* the smallest ones (between 222 and 240 bp in all species) (Fig. 2, File S5–6). The genes *Sgs7* and *Sgs8* are closely related to *Sgs3* and they can be distinguished from *Sgs3* by the length of their coding sequence (Fig. 2) and the fact that they are located at distinct genomic locations (see below).

### *Sgs1* did not duplicate and was lost at least twice via gene deletions

**Fig. 3:**
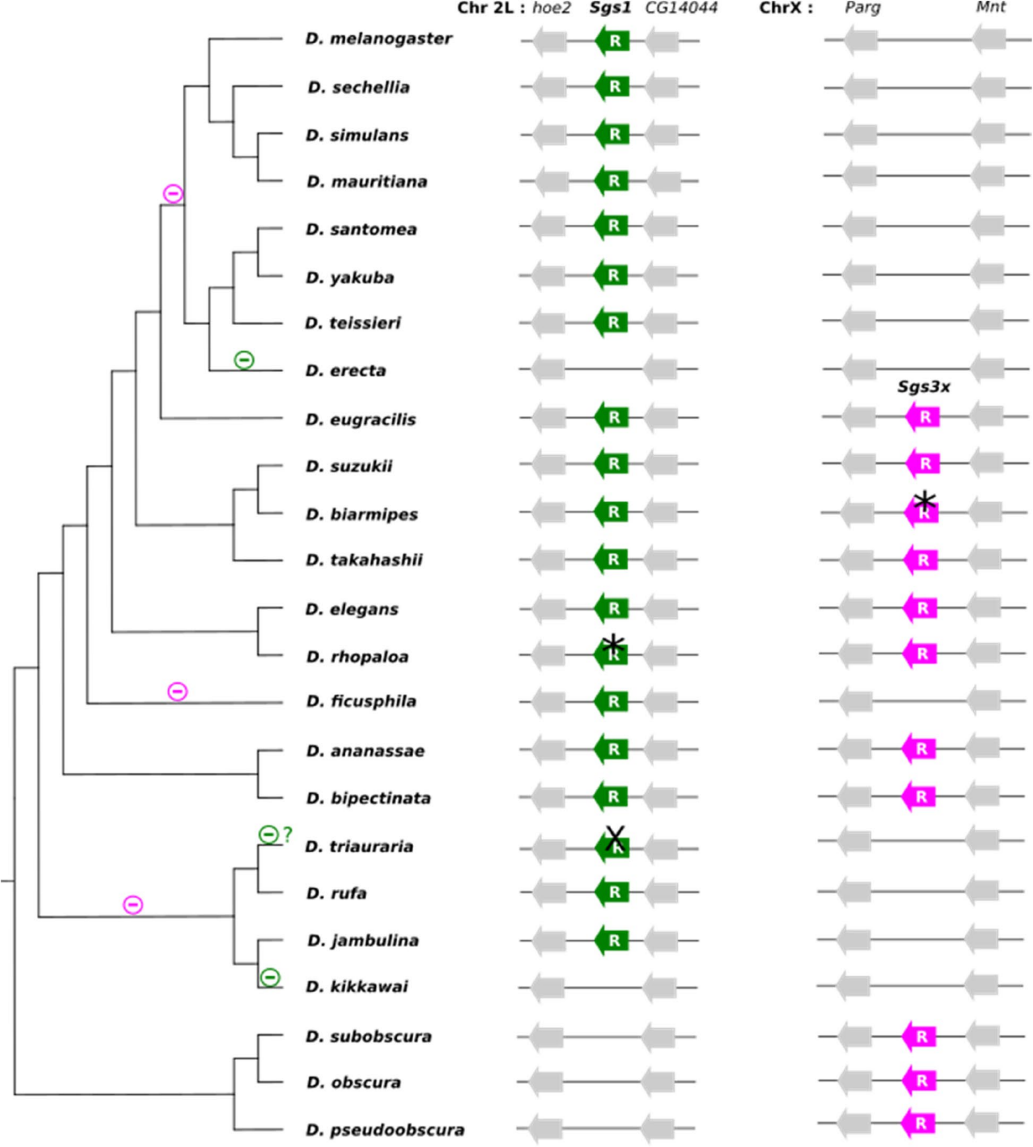
Distribution of the *Sgs1* and *Sgs3x* genes across the 24 studied Drosophila species and most parsimonious scenario for gene gains and losses. The species tree is from [33]. Branch distances are not on scale. Green, pink and gray arrows represent, respectively, *Sgs1, Sgs3x* and their adjacent neighboring genes. Gene lengths and intergenic distances are not to scale. “R” means that internal repeats are present. The cross ‘X’ on top of the *D. triauraria Sgs1* gene indicates the presence of six premature stop codons in the published genome sequence, which may be genuine stop codons or sequence assembly artifacts. * indicates a premature stop codon present in the published coding sequence of *D. rhopaloa and D. biarmipes*, which we consider as an artifact (see text for details). Minus signs on tree branches indicate gene deletion events for *Sgs1* in green and for *Sgs3x* in pink. Minus sign followed by ‘?’ indicates a presumed loss of a functional gene coding region that has not been confirmed by resequencing

**Fig. 4:**
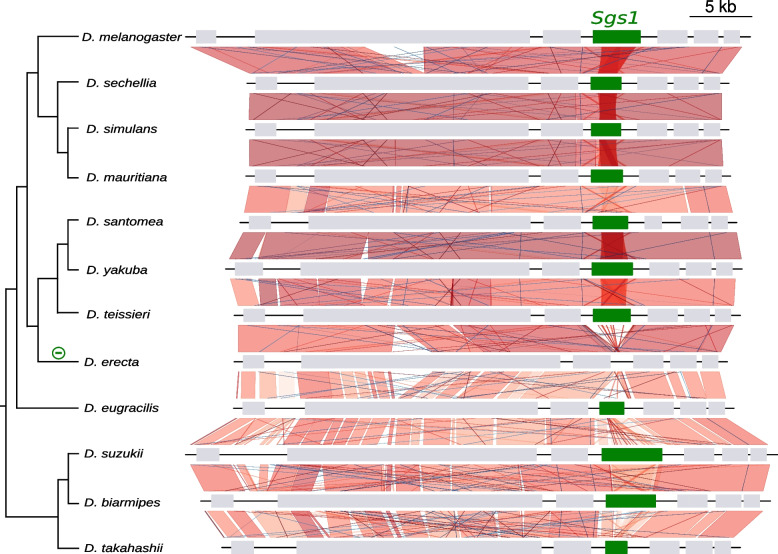
Comparison of the *Sgs1* gene region between Drosophila species closely related to *D. melanogaster*. The species tree is from [33]. Branch distances are not on scale. Boxes represent coding genes. *Sgs1* is in green and its neighboring genes in light gray. Introns and gene orientation are not shown. Vertical and diagonal lines between genomic sequences represent the pairwise similarity based on BLASTn analyses. They are red when BLASTn matches in the same direction and blue when BLASTn matches in the opposite direction. Shades of red and blue indicate the level of identity, with darker color for higher similarity. The minus sign on the tree branches indicates a gene deletion event

**Fig. 5:**
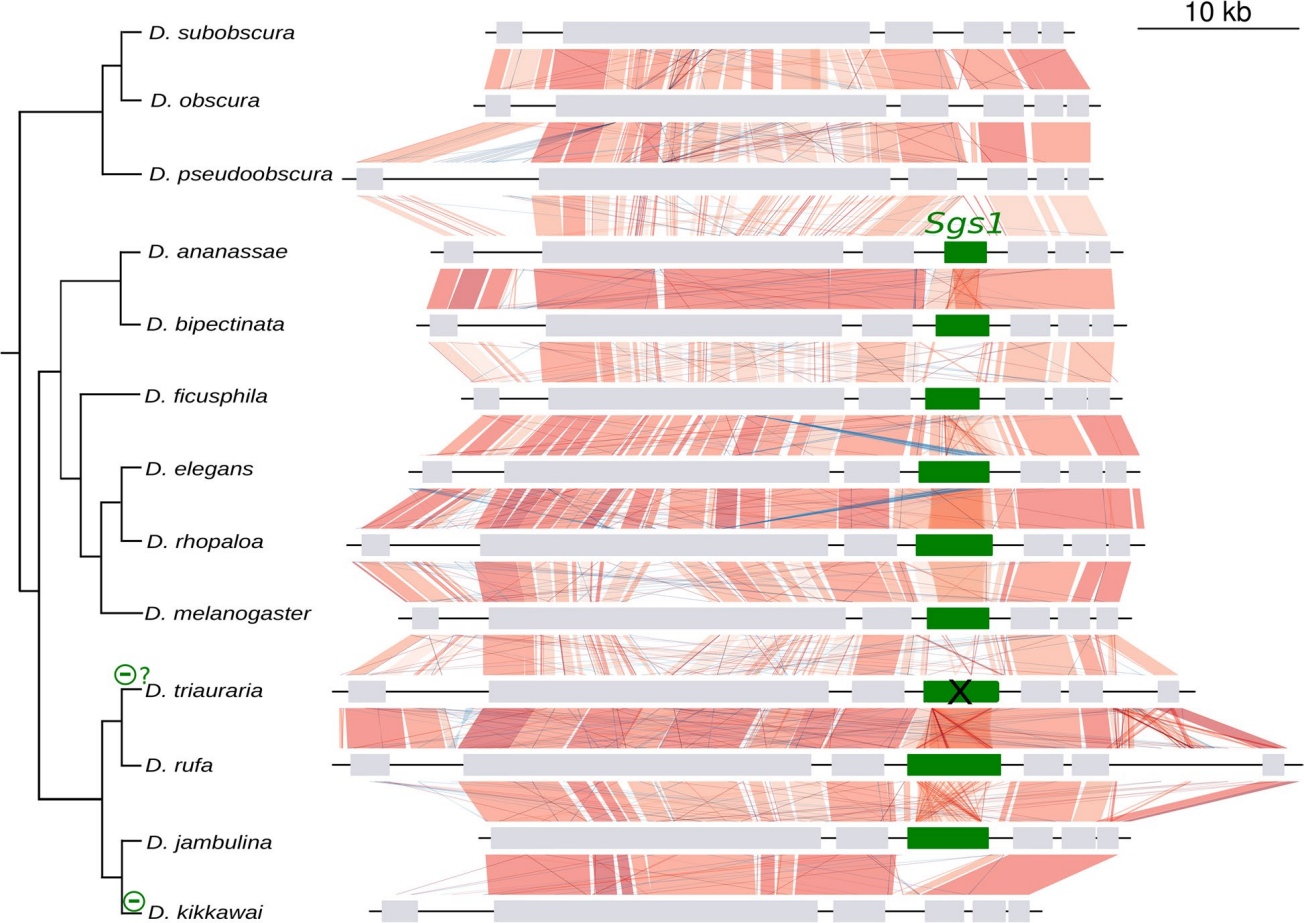
Comparison of the *Sgs1* gene region between Drosophila species. Same legend as in Fig. 4. The cross on top of the *D. triauraria Sgs1* gene indicates the presence of six premature stop codons and frameshifts in the published *Sgs1* gene sequence, which may be real or sequence assembly artifacts (see text for details)

In all the Drosophila species studied, *Sgs1* is composed of a first coding exon which is always 28 bp, a short phase 1 intron whose size varies between 50 bp and 71 bp, and a second exon which harbors a long repeat region and whose size varies from 1758 bp in *D. takahashii* to 5861 bp in *D. rufa* (Table S4). The synteny of *Sgs1* and its neighboring genes is conserved across all species (Figs. 3, 4, 5, Table S3). Using BLAST searches, *Sgs1* was not found in *D. erecta* and *D. kikkawai.* The loss of *Sgs1* in *D. erecta* and in *D. kikkawai* is associated with a 4-kb and a 3-kb deletion, respectively (according to *D. teissieri* and *D*. *jambulina* sequences, respectively), thus removing the full *Sgs1* coding region while preserving the two neighboring coding genes *hoe2* and *CG14044* (Figs. 4, 5, File S7). We conclude that two recent *Sgs1* gene losses occurred, in association with gene-wide deletions.

In the outgroup species *D. pseudoobscura, D. obscura* and *D. subobscura*, and in further distantly related species, no *Sgs1* gene was found at the syntenic location (Fig. 5) nor across the whole genome via BLAST. This suggests that the *Sgs1* gene appeared after the divergence between the most recent common ancestor of these species and *D. melanogaster*, i.e. about 30 million years ago [34]. Our analysis reveals that since its appearance within the Drosophila genus, the *Sgs1* gene has maintained the same neighboring genes throughout all the Drosophila species we examined and that it did not duplicate.

### *Sgs3x* did not duplicate and was lost at least three times via gene deletion

As for *Sgs1*, the first coding exon of *Sgs3x* is 28 bp in all the studied species and the second exon harbors repeats and varies in size, from 581 bp for *D. elegans* to 4148 bp for *D. bipectinata*. In all species featuring an *Sgs3x* gene, the gene is located at the same corresponding genomic location, between genes *Parg* (*CG2864*) and *Mnt* (*CG13316*) (Fig. 3).

**Fig. 6:**
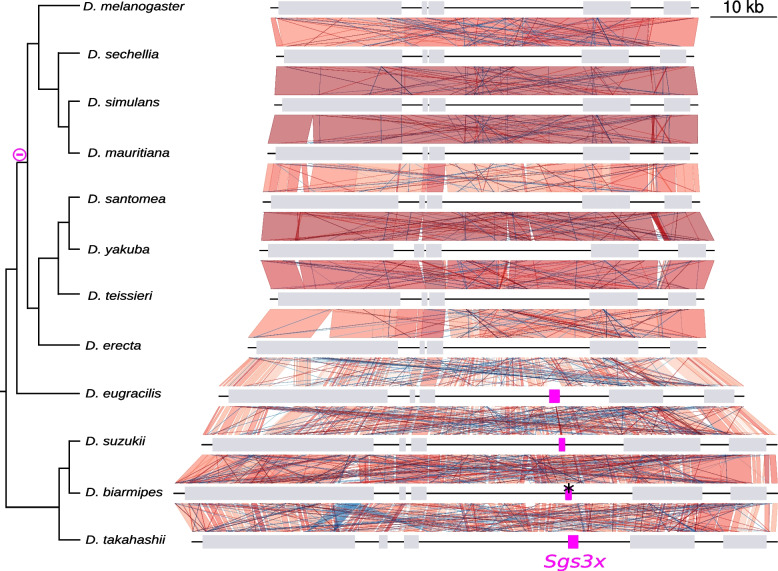
Comparison of the *Sgs3x* gene region between Drosophila species. Same legend as in Fig. 4. Pink boxes represent Sgs3x. * indicates a premature stop codon present in the published coding sequence of *D. biarmipes*, which we consider as an artifact (see text for details)

**Fig. 7:**
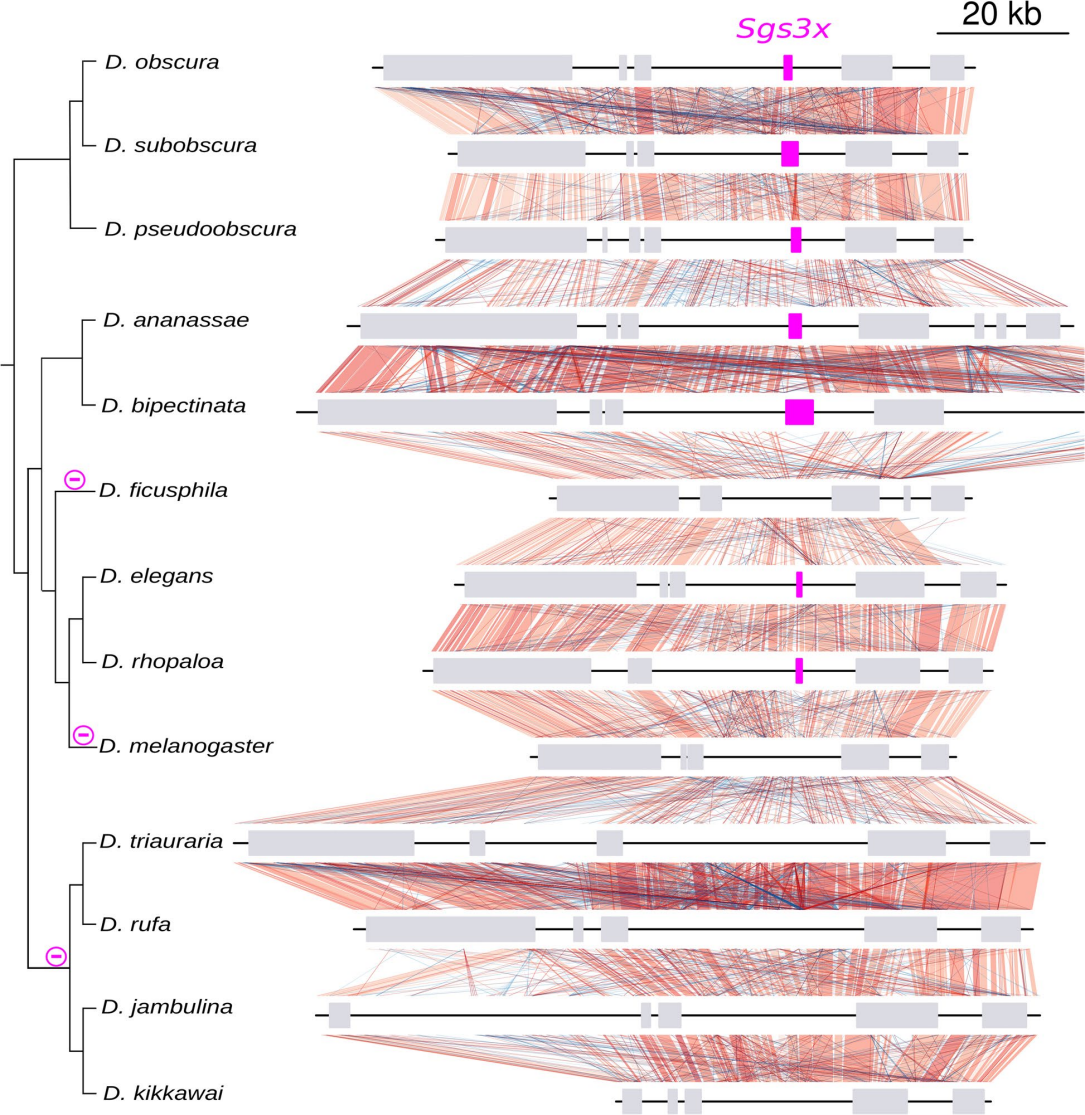
Comparison of the *Sgs3x* gene region between distantly related Drosophila species. Same legend as in Fig. 4. Pink boxes represent *Sgs3x.* Part of the genomic region of *D. bipectinata* (right) has been cut for clarity

The most parsimonious scenario is that *Sgs3x* was already present in one copy in the ancestor of the species studied here. Based on our phylogenetic analysis and parsimony, we infer that *Sgs3x* has been lost three times: before the most recent common ancestor of *D. melanogaster* and *D. erecta* (*melanogaster* subgroup) (Fig. 6, via a 1-kb deletion when compared with *D. eugracilis),* in the ancestor of *D. triauraria, D. rufa, D. jambulina* and *D. kikkawai* (*montium* group) (Fig. 7, via a 2-kb deletion compared to *D. bipectinata*) and in the ancestor of *D. ficusphila* (Fig. 7, via a 1-kb deletion compared to *D. elegans).* Overall, *Sgs3x* exhibits an evolutionary history like *Sgs1*: it did not change neighboring genes, did not duplicate and experienced deletions of its full gene coding sequence in a few species.

### Two *Sgs3* copies lost their internal repeats in the lineage leading to *D. subobscura*

**Fig. 8:**
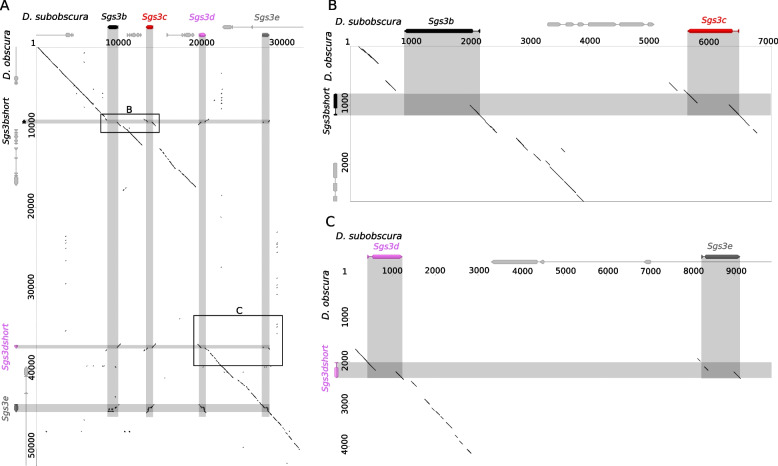
Dot plot comparing *D. subobscura* and *D. obscura Sgs3* genomic regions*.* **A** Main dot plot. **B**-**C** Magnifications of the regions of interest indicated in (**A**). Black diagonal lines indicate matching genomic regions. Black, red, pink, and dark gray arrows represent, respectively, *Sgs3b, Sgs3c, Sgs3d* and *Sgs3e.* Light gray arrows represent neighboring genes. Numbers indicate nucleotide positions in bp

**Fig. 9:**
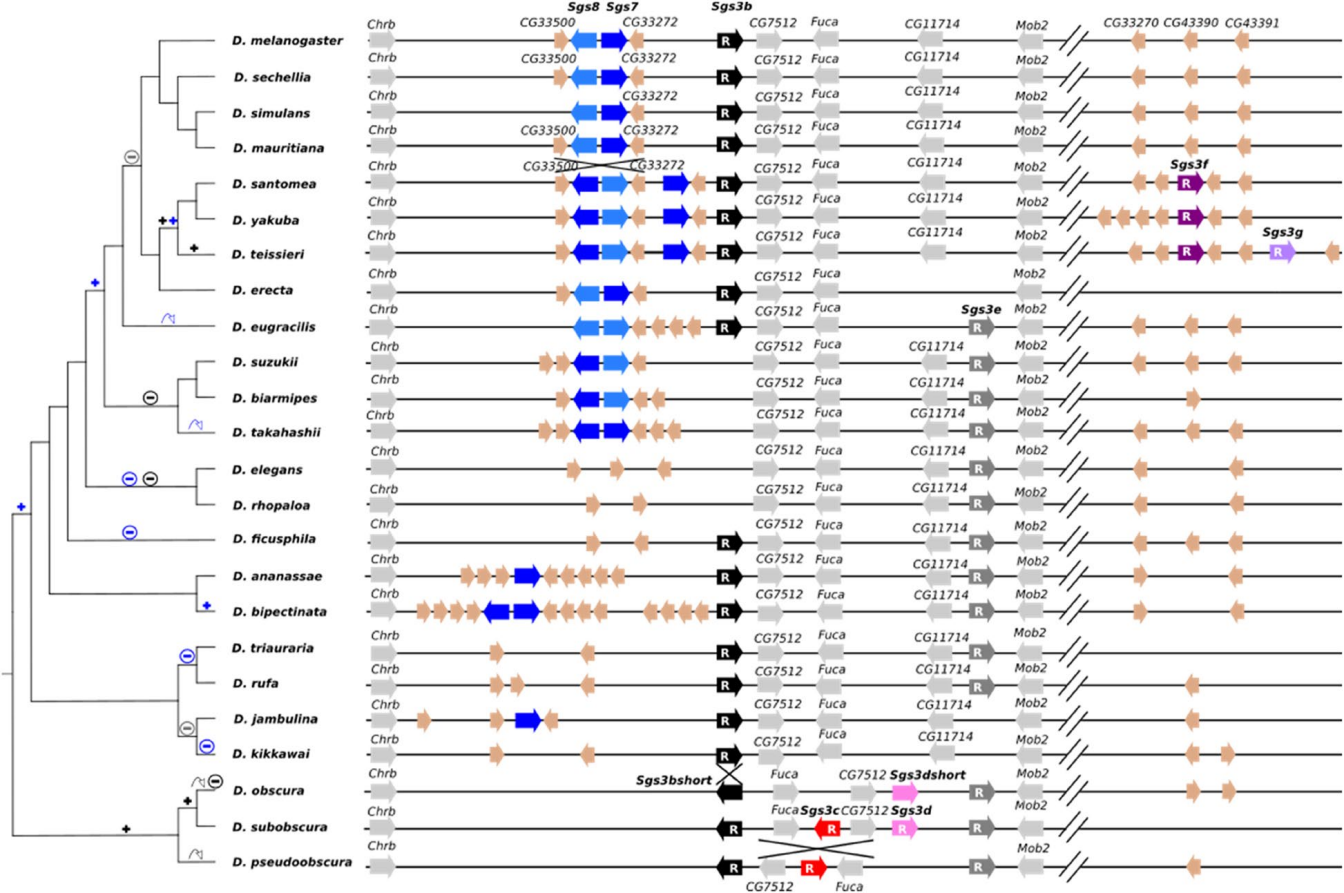
Distribution of the *Sgs3, Sgs7* and *Sgs8* ortholog genes across the 24 studied Drosophila species and the most parsimonious scenario for gene gains and losses. Same legend as in Fig. 3. Black, red, pink, dark gray, dark purple and light purple arrows represent different copies of *Sgs3* (respectively *Sgs3b, Sgs3c, Sgs3d, Sgs3e, Sgs3f, Sgs3g*). Dark blue and light blue arrows represent *Sgs7* and *Sgs8,* respectively. Here we present one proposition for the attribution of the names *Sgs7* and *Sgs8* to the short *Sgs* glue genes, but the distinction between *Sgs7* and *Sgs8* can be unclear. Beige arrows indicate genes encoding short threonine-rich proteins. Light gray arrows indicate other adjacent neighboring genes. The *Sgs3e* coding sequence is located within *Mob2* intron, but is represented near *Mob2* for simplicity*.* Also note that for clarity a few of the neighboring genes and their corresponding orthologs were omitted in this figure. Arrows, minus and plus signs on the tree branches indicate, respectively, gene conversion, gene deletion and duplication events for *Sgs3* in black and for *Sgs7* and *Sgs8* in blue. Crosses designate inversions. Double lines interrupting the genomic sequence indicate a gap of about 50 kb. Here we assumed that the most recent common ancestor of all represented species had two *Sgs3* copies, *Sgs3b* and *Sgs3e*

We define *Sgs3, Sgs7* and *Sgs8* as copies of the *Sgs1-Sgs3-Sgs7-Sgs8* gene family that are present within a large genomic syntenic block corresponding to region 68C10–11 on chromosome 3 L *in D. melanogaster.* The *Sgs3* genes are distinguished from *Sgs7* and *Sgs8* by the presence of repeats and by longer coding regions (Fig. 2). However, in *D. obscura,* at the loci occupied by *Sgs3b* and *Sgs3d* in *D. subobscura,* we detected two *Sgs3* genes which are shorter (both 270 bp) than typical *Sgs3* genes (Fig. 2), do not present internal repeats but share similar N-terminal and C-terminal regions with their corresponding *Sgs3* copies in *D. subobscura* (Fig. 8). Dot plots suggest that the repeated sequences of *Sgs3b* and *Sgs3d* were lost in the lineage leading to *D. obscura* (Figs. 8, 9)*.* We named the resulting genes in *D. obscura Sgs3bshort* and *Sgs3dshort.* The coding sequence of these two genes are extremely similar (Fig. 1B), suggesting that they originate from a recent gene conversion event in the lineage leading to *D. obscura* (Fig. S3–4). In addition to *Sgs3bshort* and *Sgs3dshort*, *D. obscura* possesses a copy of *Sgs3e* harboring internal repeats (Figs. 8, 9). Complete losses of internal repeats were not observed in *Sgs1* nor in *Sgs3x* (Table 1).

**Table 1:** Summary of the sequence changes observed for the different *Sgs* gene subfamilies in the 24 studied species. Numbers indicate the number of genetic events inferred for each *Sgs* gene

	*Sgs1*	*Sgs3x*	*Sgs3e*	*Sgs3b*	*Sgs7-Sgs8*
inferred number of copies in the common ancestor of all studied species	0 (appeared after the *D. melanogaster/D. pseudoobscura* divergence)	1	1	1	0 (appeared after the *D. melanogaster/D. pseudoobscura* divergence)
position and orientation relative to neighboring genes	constant	constant	constant	variable	variable
first coding exon size	constant (28 bp)	constant (28 bp)	variable (19-28 bp)	variable (25-31 bp)	constant (28 bp)
internal repeats	present	present	typically present	typically present	typically absent
loss of all the internal repeats	0	0	0	2	not applicable
gene deletion	2	3	2	2	4
gene duplication	0	0	0	4	≥ 3
gene inversion	0	0	0	2	≥ 1
gene conversion	0	0	0	2	≥ 3

### *Sgs3* underwent several duplications, deletions, inversions and gene conversions

As opposed to *Sgs1* and *Sgs3x, Sgs3* first exon varies slightly in size, from 19 bp to 28 bp (Table S4). The second exon length varies from 356 bp in *D. jambulina Sgs3b* to 1967 bp in *D. bipectinata Sgs3e* (Table S4). The beginning of the second exon of *Sgs3* encodes for a relatively conserved amino acid sequence, ASILLI (Fig. 1A). Two *Sgs3* copies are found in most of the studied species: *Sgs3b* (which is located between genes *CG33272* and *CG7512*) and *Sgs3e* (which is located within an intron of the gene *Mob2*) (Fig. 9, S4). Parsimony suggests that both genes were present in the most recent common ancestor of all studied species (Table 1). Comparison of protein sequences (File S8) shows that *Sgs3c*, *Sgs3d*, *Sgs3f* and *Sgs3g* are duplicates of *Sgs3b* and that *Sgs3e* did not duplicate in the lineages studied here. The high similarity between the two *Sgs3* copies present in *D. pseudoobscura* is also indicative of gene conversion. Parsimony principle indicates that across the 24 studied species, *Sgs3e* underwent 2 gene losses and no duplications whereas *Sgs3b* experienced 2 gene losses and 4 gene duplications, all within the same syntenic block (Fig. 9, Table 1). Furthermore, inversions of the entire *Sgs3* coding sequence, together with adjacent regions, occurred in two instances (crosses in Fig. 9, S5). Such inversions were not observed for *Sgs1* nor for *Sgs3x* (Table 1).

### *Sgs7* and *Sgs8* underwent several duplications, gene losses and gene conversion

*D. melanogaster* possesses two glue genes near *Sgs3b* that are devoid of internal repeats, *Sgs7* and *Sgs8*. In the other 23 Drosophila species, we annotated in the corresponding syntenic region 0, 1, 2 or 3 *Sgs* genes with no repeats (Fig. 9). For all these *Sgs7* and *Sgs8* orthologs, the size of the first coding exon is 28 bp and the second coding exon size varies between 194 bp in *D. ananassae Sgs7* and 212 bp in *D. bipectinata Sgs7b*.

**Fig. 10:**
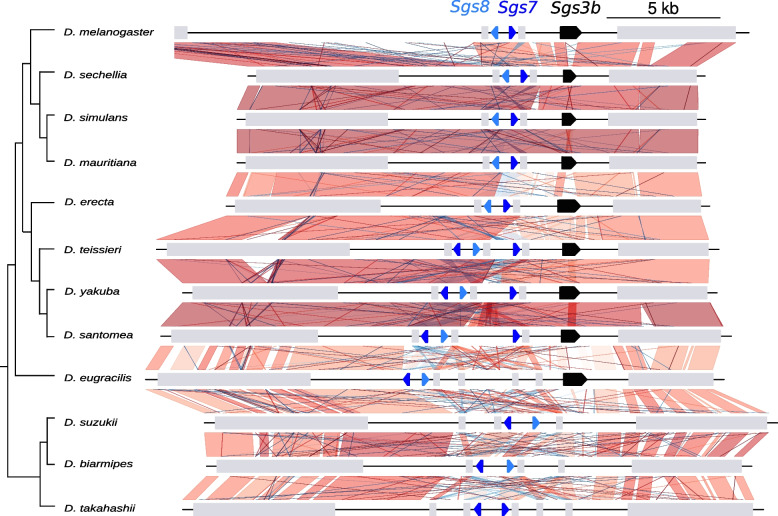
Closer view of the comparison of the *Sgs3–7-8* gene region between Drosophila species. Same legend as in Fig. 5. *Sgs7* copies are in dark blue, *Sgs8* in light blue. Note that our distinction between *Sgs7* and *Sgs8* is subject to caution (see text for details). *Sgs3b* is represented in black. *Sgs* genes directions are given by arrows. Neighboring genes directions are not shown

The two *Sgs8* copies in *D. eugracilis* exhibit very similar sequences (Fig. S6), suggesting that they originated from a recent duplication or from gene conversion in the branch leading to *D. eugracilis* (Fig. 9). Similarly, another recent duplication or gene conversion event seems to have occurred in the branch leading to *D. takahashii* (Figs. 9, 10). In certain cases, it was impossible to determine with absolute confidence whether the different copies correspond to *Sgs7* or *Sgs8*, due to their short coding sequences, their rapid divergence and signs of gene conversion. For example, *D. erecta* and *D. teissieri* harbor *Sgs* genes at the exact genomic positions corresponding to *D. melanogaster Sgs7* and *Sgs8* genes (Fig. 10). However, at the *Sgs7* position in *D. teissieri* is a coding region which is closer to *Sgs8* than *Sgs7*, and reciprocally at the *Sgs8* position (Fig. 1B). Dot plot analysis (Fig. S7) suggests that gene conversion occurred between *Sgs7* and *Sgs8* in the lineage leading to *D. teissieri*. Overall, our distinctions between the *Sgs7* and *Sgs8* genes are thus subject to caution.

In addition, synteny comparisons suggest that an inversion occurred between the group of *D. santomea*, *D. yakuba*, *D. teissieri* and *D. erecta*, and the *melanogaster* complex (*D. melanogaster*, *D. simulans*, *D. sechellia* and *D. mauritiana*), which inverted a pair of *Sgs7* and *Sgs8* genes together with their adjacent genes (Figs. 9, 10, S8). And further gene conversion events blurred the relationships between *Sgs7* and *Sgs8* in these four species (Figs. 9, 10, S8).

In summary, a single copy of *Sgs7*–8 was probably present in the common ancestor of *D. kikkawai* and *D. melanogaster*. It underwent at least 4 deletions, 3 duplications, one inversion and several gene conversion events (Table 1).

### Genomic instability is associated with the presence of short “new glue” genes

Our analysis reveals two types of gene dynamics. A first group of genes, comprising *Sgs1*, *Sgs3x* and *Sgs3e,* experienced several gene losses but no duplication, no local inversion and no gene conversion across the 24 Drosophila species studied here. In contrast, the second category, involving *Sgs3b*, *Sgs7* and *Sgs8*, underwent multiple events of duplication, local inversion and gene conversion (Table 1, Fig. 9).

To test the potential involvement of repetitive elements, we looked for the presence of repeated sequences across 129-kb regions encompassing each *Sgs* gene in several Drosophila species (Fig. S9). We found that in *D. melanogaster* repeats are more frequent near the *Sgs3b/Sgs7/Sgs8* genes than around the *Sgs1* and *Sgs3x* genes*.* Furthermore, the recently duplicated genes *Sgs3c* and *Sgs3d* in *D. subobscura* and *Sgs3f* and *Sgs3g* in *D. teissieri* locate within regions dense in repeats. Interestingly, multiple genomic changes (duplications, inversions) were found at the *Sgs7–8-3b* and *Sgs3f-g* loci, and similar stretches of sequences were detected at both loci (Fig. S10). These sequences contain short (243–426 bp), intronless genes encoding for threonine-rich proteins with predicted signal peptides. These genes resemble four genes adjacent to *Sgs4* that were previously annotated in *D. melanogaster* as “nested genes” or “new glue genes”, even though their putative role in glue production is unclear [35, 36] (Fig. S11). We thus decided to name the new sequences we identified as *new glue* (*ng)* genes.

In total, we annotated 154 such *ng* genes in the *Sgs3–7-8* genomic region of the 24 studied Drosophila species (Table 2, S3). We define *ng* genes as encoding for proteins displaying the following characteristics: (1) a protein shorter than 180 amino acids, (2) a signal peptide, (3) an internal region rich in alanines and containing stretches of at least three consecutive threonines, and (4) a C-terminal region rich in arginines and lysines (Fig. S11). The previously annotated *ng4* gene from *D. melanogaster* does not exhibit characteristics (2) to (4). The threonine stretch can attain up to 17 consecutive threonines, as in *D. ananassae LOC6500299*. Noticeably, almost all the *Sgs7* and *Sgs8* genes are adjacent and tail-to-tail to an *ng* gene, with approximately 130–200 bp separating the stop codons of both genes (beige arrows in Fig. 9). *Sgs3f* and *Sgs3g* are distant of approximately 400 bp from their tail-to-tail adjacent *ng* gene. Most duplications and inversion events appear to preserve the contiguity and distance between the *Sgs* gene and its adjacent *ng* gene (Fig. S12-S14).

**Table 2:** Number of *ng* genes identified in 7 representative species (*D. melanogaster, D.ananassae, D. obscura, D. subobscura, D. willistoni* and *D. virilis*)*.* Each column corresponds to a genomic region. Note that the 87A1 locus is located 5 Mb away from *Sgs5* and that the 3C11–12 locus is 500 kb away from *Sgs1* in *D. melanogaster.* No *ng* gene was found near *Sgs1, Sgs3e* and *Sgs3x*

Species	3C11–12 (near *Sgs4, Notch* and *dnc*)	68C11 (near *Sgs3b, Sgs7, Sgs8*)	68C13 (near *Sgs3f, Sgs3g*)	28E6-28E7 (near *mon2, Bsg* and *CG8673)*	87A1 (near *cad87A, CG6959* and *sad)*	88C3–4 (near *Cystatin-like, Phosphodiesterase 6* and *stumps*)
*D. melanogaster*	4	2	4	none	none	4
*D. ananassae*	none	8	4	none	10	4
*D. obscura*	6	none	2	none	none	1
*D. subobscura*	5	none	none	none	none	3
*D. willistoni*	none	none	none	2	none	2
*D. virilis*	none	none	none	none	none	none
*D. hydei*	none	none	none	none	none	none

We used BLAST to search for *ng* genes in other parts of the genome and we identified three additional loci, containing *ng* genes but no *Sgs* genes, in several of the 24 studied species (Table 2). In *D. melanogaster*, two of these three loci (87A1 and 88C3–4) are separated from each other by approximately 2 Mb. No *ng* gene was found at the *Sgs1* and *Sgs3x* loci. Furthermore, no *ng* genes were detected by BLAST in the full genomes of *D. virilis* and *D. hydei*. This suggests that *ng* genes appeared after the divergence of *D. virilis* and *D. melanogaster*.

In summary, a family of new genes called “new glue” genes was detected near *Sgs* genes in highly dynamic regions (*Sgs7–8-3b* and *Sgs3f-g*), but not in less dynamic regions (*Sgs1* and *Sgs3x*).

### A recent gene duplication and an inversion were probably mediated by new glue genes

**Fig. 11:**
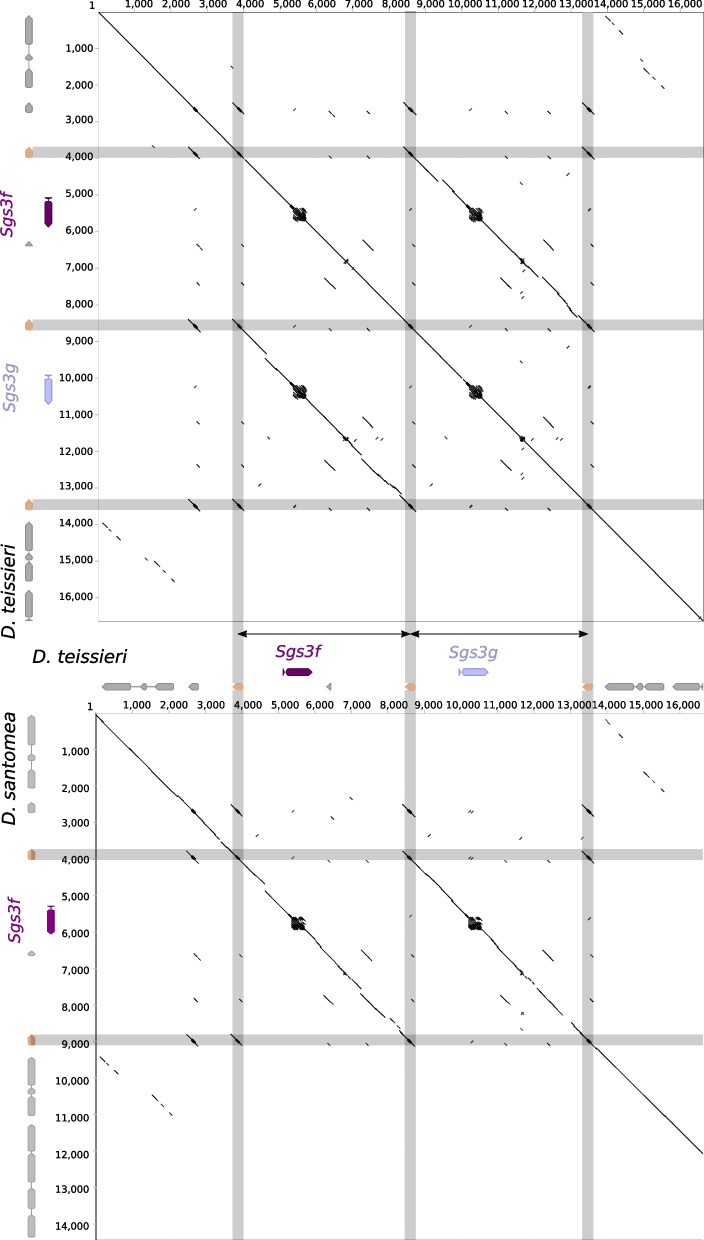
Dot plots of genomic regions from *D. santomea* and *D. teissieri.* In the upper dotplot, *D.teissieri Sgs3f/Sgs3g* genomic region is compared to itself. In the lower dotplot, *D.teissieri Sgs3f/Sgs3g* genomic region is compared to *D. santomea Sgsf* genomic region. Dark and light purple arrows represent *Sgs3f* and *Sgs3g,* respectively*.* Grey arrows represent neighboring genes. Beige arrows represent *ng* genes located at the duplication breakpoints. Double-headed black arrows indicate the duplicated region

To investigate whether these new glue genes may have played a role in the evolutionary dynamics of genomic regions, we examined whether they were present at the boundaries of three relatively recent genomic rearrangements. First, we found that the duplication leading to *Sgs3d* in *D. subobscura* (which likely occurred approximately 15 million years ago [34]) (Fig. 9) included 5′ and 3′ non-coding regions surrounding the *Sgs3b* gene, and that there were no *ng* genes in the region (Fig. S15). Second, for the inversion of the *Sgs7-Sgs8* region which occurred just before the divergence of *D. teissieri* and *D. santomea* (around 2–11 million years ago [34]) (Fig. 9), we noticed that one of the breakpoints perfectly corresponds to the coding region of a *ng* gene (Fig. 11). Third, for the recent duplication leading to *Sgs3g* in *D. teissieri* (which occurred about 0–2 million years ago [34]), both breakpoints corresponded to *ng* genes (Fig. 11). The older the event, the more likely sequences at the breakpoints may be lost or modified. Here, we found that two breakpoints of a recent gene duplication and one breakpoint of an older inversion match the coding regions of *ng* genes. Given that *ng* genes are found in multiple copies over the genome, we suggest that they may facilitate large-scale genomic modifications such as gene inversion, gene duplications and gene losses.

## Discussion

We reconstructed the evolutionary history of 102 *Sgs* genes present in 24 Drosophila species, including 26 newly annotated *Sgs* genes. Compared to our previous Da Lage et al. 2019 study [25], we used higher quality genome assemblies, synteny comparisons and blast queries from multiple species. This strategy allowed us to identify 13 new *Sgs* genes not reported in Da Lage et al. The *Sgs* glue genes can be difficult to annotate because their coding region is mostly composed of large repetitive sequences (prone to sequence misassembly and frameshifts) and evolves rapidly [23, 25]. We propose here a new nomenclature for *Sgs* genes based on protein sequence conservation, genomic location and presence/absence of internal repeats.

Our analysis suggests that three *Sgs* genes (*Sgs3x*, *Sgs3b*, *Sgs3e*) were probably present in the most recent common ancestor of all studied species and that the *Sgs1* and *Sgs7/8* genes arose after the divergence between *D. pseudoobscura* and *D. melanogaster*, i.e. about 30 million years ago [34]. No clear homologs of *Sgs1* and *Sgs7/8* were detected in more distantly related species using BLAST or HMMER, so the origin of these genes remain unclear.

The Sgs1 proteins exhibit a highly conserved motif, PCPC-X(1)-PQPP (Fig. 1A) which is also found in an uncharacterized domain of Suppressor of cytokine signaling 7 protein in mouse and human according to Prosite searches. The conserved motif C-x(2)-CGPGG from *Sgs3/7/8/3X* is found in the hormone transporter neurophysin in several mammal species and one mollusc. Interestingly, part of this sequence is also found in the repeat motifs (GGX or GPGXX) present in several silk proteins from spiders [37]. These stretches of amino acids probably evolved by convergent evolution in these proteins and in glue proteins.

Our present analysis of 24 Drosophila species spanning approximately 30 million years of evolution reveals that the *Sgs1, Sgs3x* and *Sgs3e* genes have remained at the same exact genomic location relative to their neighboring genes and did not duplicate, whereas the other genes (*Sgs3b*, *Sgs7, Sgs8*) have experienced inversions, translocations and duplications. Our observations are in agreement with a 1986 study which compared sequences from 5 closely related species of Drosophila and detected a 6-kb region containing *Sgs3*, *Ssg7* and Sgs8 which evolved faster than neighboring regions, via point mutations, insertions, deletions, inversions and the gain and loss of repetitive sequences [38]. In our study we did not assess mutation rate within coding sequences nor intraspecific variation.

In *D. virilis*, which diverged about 43 millions years ago from *D. melanogaster* [34] and was not examined in this study, three glue genes have been identified: *Sgs3a/Lgp1*, *Sgs3b/Lgp3* and *Sgs5bis/Lgp2* [24]. *Sgs* gene sequence divergence is too large between *D. virilis* and the species analyzed in this study to rely on phylogenetic trees to infer the relationship between their glue genes. *Sgs3a/Lgp1* and *Sgs3b/Lgp3* are adjacent to each other and result from a recent duplication in the *D. virilis* lineage [25]. Both genes lie near *AstA-R1, Ilp7, Parg* and *Rala* genes, which are also located at the *Sgs3x* locus in the species studied here. This suggests that *Sgs3a/Lgp1* and *Sgs3b/Lgp3* in *D. virilis* correspond to *Sgs3x* orthologs and that a gene duplication affecting *Sgs3x* did occur in species outside of the range of Drosophila species studied here.

Studies of *D. melanogaster Sgs1*, *Sgs3*, *Sgs4, Sgs5, Sgs7* and *Sgs8* indicate that glue genes display short, compact cis-regulatory regions that directly flank their start codon (within less than 1–2 kb) [39–44]. Such a characteristic, as observed for odorant receptor genes in insects [45], may facilitate gene turnover as shuffling of genomic regions is less likely to disrupt gene regulation. The *Sgs* genes we studied here display comparable expression patterns and amino acid sequences [24], so their difference in gene turnover dynamics does not seem to be related to variation in their gene function. Here we investigated the possible role of genomic context on glue gene dynamics. We observed that regions with high *Sgs* gene turnover contain copies of short coding genes named *new glue* (*ng*) genes that are immediately adjacent to the *Sgs* genes, whereas regions with low *Sgs* gene turnover do not. Several pieces of evidence suggest that the presence of these flanking *ng* genes may accelerate gene dynamics: they are usually found in multiple copies at specific genomic locations, they lie near glue genes with rapid gene dynamics but not near the ones with reduced gene dynamics, they locate at two breakpoints of a recent *Sgs* gene duplication (0–2 million years ago) and at one breakpoint of an older inversion. These *ng* genes provide regions of high sequence identity for homologous recombination and thus may trigger genomic instability, similarly to the indirect effect of transposable elements on genome dynamics [12]. Interestingly, we detected no increase in the rate of gene evolution for the other genes located near the *ng* genes in the *Sgs3b-7-8* region. For example, the genes *CG7512, Chrb* and *Mob2* did not undergo any local duplication or deletion across the studied species (Fig. 9). It is possible that genomic instability is particularly strong for glue genes because their internal repeats and signal peptide sequences can match the ones present within the *ng* genes and thus trigger genomic rearrangements.

The 4 *ng* genes near *Sgs4* were first named “*nested genes*” (abbreviated as “*ng*”) because they are nested together with *Sgs4* within the intron of the unrelated phosphodiesterase gene *dunce* [35, 36]. Three of them were found to resemble *Sgs3,* except that the intron was missing and the internal repeat region was smaller [35]. In the following publications, their name became “ng glue” [46] and then “new glue” [47, 48], with no justification given. In this study, we follow the most recent nomenclature and name them “new glue” (*ng*) genes, even though we are aware that no functional study has been reported so far to test the hypothesis that they are involved in glue production or adhesion. We identified 154 *ng* genes in 24 Drosophila species. 89 of them are newly annotated genes that were not identified previously. The *ng* genes can be difficult to annotate because they appear to evolve rapidly and they are small genes (and thus may not generate sufficiently significant E-values in BLAST searches).

Our study reveals that *ng* genes surround not only *Sgs4* but also *Sgs3b/f/g*, *Sgs7* and *Sgs8* (Table 1). It would be interesting to examine the evolutionary dynamics of *Sgs4* genes to test whether the presence of neighboring *ng* genes might also promote genome dynamics at the *Sgs4* locus. In *D. melanogaster, ng* genes are found in at least four genomic locations and the expression pattern of 3 *ng* genes (*ng-1*, *ng-2* and *ng-3*) has been thoroughly studied in the 1990s. These three genes are exclusively expressed within the larval salivary glands [36] and only during a short temporal window, from the beginning of the third larval instar until the early wandering stage [49]. Proteins encoded by some of the *ng* genes have also been detected in a proteomics study in the whole body of developing larvae [50]. The presence of a putative signal peptide and an internal region rich in threonines (putative glycosylation sites) indicate that they may encode proteins that participate in the production of the glue. The presence of active ecdysone-responsive elements detected with the coding regions of *ng-1*, *ng-2* and *ng-3* [51, 52] also suggest that part of their function might be related to the regulation of expression of the neighboring glue genes. Several RNAi lines are available for future work to assess the role of *ng* genes in glue production and glue adhesiveness.

During animal evolution various glands evolved to produce large amounts of very specific proteins with diverse functions, such as venom in snakes and frogs or silk in spiders [53, 54]. Recent evolutionary studies indicate that, similarly to Drosophila glue genes, the genes encoding these secreted proteins underwent multiple events of gene duplications, losses and conversions in snakes and spiders [55, 56]. Our work on Drosophila glue genes, in combination with studies of these other secretory fluids, may thus help to provide general insights on how secretory products rapidly adapt to biotic and abiotic factors.

## Conclusions

In this study, we used comparative phylogenomic methods to identify and characterize glue genes that are rapidly evolving in Drosophila species to better understand their dynamics in terms of duplications, losses, inversions and gene conversions. We uncovered several “glue” and “new glue” genes that were not found in previous studies and we propose a new nomenclature for glue genes. Our work highlights two modes of evolution for glue genes, differing in rates of inversion, duplication, gene loss and conversion. The most dynamic genes (*Sgs3b*, *Sgs7* and *Sgs8*) are in a region containing multiple “new glue” genes. Our analysis suggests that the presence of these short genes may have contributed to the higher dynamics of glue genes in this region. Our results serve as a framework for future studies on glue genes and glue adhesion in Diptera flies. This work also reveals new avenues of research for understanding why certain genomic regions evolve faster than others.

## Methods

### Fly stocks and nucleic acid extraction

To amplify part of the *Sgs1* gene, we used the following stocks: *D. rhopaloa* (line BaVi067 from Vietnam, Hanoi Ba Vì, near Vân Hòa [21°04′N, 105°22′E], collected in March 2005, gift from N. Gompel, obtained from H. Takamori), *D. takahashii* (stock number 14022–0311.07, isofemale line from Ulu Temburong National Park, Brunei, 2003, gift from N. Gompel). Flies were cultured at 22 °C in plastic vials on standard medium [4 l: 83.5 g yeast, 335.0 g cornmeal, 40.0 g agar, 233.5 g saccharose, 67.0 ml Moldex, 6.0 ml propionic acid]. For both species, DNA was extracted from five adults (3 males and 2 females) using Omega Bio-tek E.Z.N.A. Insect DNA Isolation Kit following the manufacturer’s instructions. RNA was extracted from five adults (3 males and 2 females) using a Nucleospin RNA kit from Macherey-Nagel following manufacturer’s instructions.

### PCR and RT-PCR

For *D. rhopaloa,* Omega Bio-tek E.Z.N.A. Insect DNA Isolation Kit was used for genomic DNA extraction. We used the following primers within the *Sgs1* repeated region and framing the observed frameshift: forward 5′ ACT TGC ACC CCT CCC CCT GT 3′ and reverse 5′ GGA GTG CAC CCC AAC GCG AT 3′. The primer set gave a smear or shorter fragments than expected at different PCR conditions using Phusion high fidelity DNA polymerase (New England Biolabs, M0530S). We conclude that the repeated region where the primers were designed in *D. rhopaloa Sgs1* region did not allow us to successfully amplify the region of interest. Primer sets outside of the repeated region could not be used for PCR since the repeated region is close to 5 kb.

For *D. takahashii* and *D. rhopaloa,* RNA was extracted from three third instar wandering larvae with Macherey Nagel Nucleospin RNA kit. A reverse transcription was then performed with the SuperScript VILO cDNA synthesis kit from Invitrogen. 200 ng of RNA were used for a reaction of 20uL. The samples were then placed 10 minutes at 25 °C, 60 minutes at 42 °C and 5 minutes at 85 °C. PCR was then performed with Gotaq from Promega. For *D. takahashii,* the following primers were used to amplify part of the *Sgs1* sequence: forward 5′ CCC GAT CCA ATG GAG CCC TGT 3′ and reverse 5′ GTG TCG GTG GCT GTG TCT GTA 3′. Annealing was performed at 55 °C. The primers amplified a 350-bp fragment which contains an extra ‘A’ nucleotide in the repeated region compared to the NCBI *D. takahashii* genome sequence (accession number GCA_000224235.2). For *D. rhopaloa,* the following primers were used: forward 5′ CCA CTC CTA CCC CCA TAA CT 3′ and reverse 5′ GGG TAG GAG TGG ATG TAG GT 3′. We obtained a smear and made the same conclusion as with the PCR results. We performed a new PCR on cDNA of *D. rhopaloa* with primers: forward 5′ ACT TGC ACC CCT CCC CCT GT 3′ and reverse 5′ GGA GTG CAC CCC AAC GCG AT 3′ (same primers as we used at first), and purified highest PCR product among several, about 5000 bp long using Nucleospin Gel and PCR cleanup kit from Macherey Nagel. We did not manage to clone and sequence the purified product.

### Annotation of *Sgs* genes

Sequence databases were searched by blastn and tblastn in a recursive manner, using the *Sgs* sequences of various Drosophila species. BLAST searches were performed via the NCBI BLAST page (https://blast.ncbi.nlm.nih.gov/Blast.cgi), the SpottedWingFlybase website (http://spottedwingflybase.org/) for *D. suzukii* or using Megablast, a variation on blastn that is faster but only finds matches with high similarity, in Geneious Prime (2019.2.3 Build 2019-09-24 10:49, Java Version 11.0.3 + 7 (64 bit)) (https://www.geneious.com/) after uploading the genomes. The coding regions were annotated manually (Table S2), using sequence homology with closely related species, conserved intron-exon structure and conserved stretches of amino acids (Fig. 1A). Peptide signals were predicted using SignalP-6.0 website (last accessed on 2022/08/24, https://services.healthtech.dtu.dk/service.php?SignalP). Annotations were then verified based on alignments of the respective protein sequences using MUSCLE (3.8.425) [57] implemented in Geneious Prime (version 2019.2.3) (https://www.geneious.com/).

Our analysis allowed us to identify 13 additional *Sgs* genes in the species previously examined by Da Lage et al.: *Sgs1* in *D. ananassae* and *D. bipectinata*; Sgs3x in *D. pseudoobscura*, *D. eugracilis*, *D. suzukii* and *D. takahashii; Sgs3* orthologs in *D. suzukii*, *D. santomea*, *D. yakuba*, *D. bipectinata*, *D. ananassae, Sgs7* in *D. ananassae* and *Sgs8* in *D. mauritiana*. We also annotated a few *Sgs* coding sequences that were absent in NCBI annotated genomes: *Sgs3e* in *D. suzukii, D. ananassae, D. eugracilis, D. takahashii, D. biarmipes, D. ananassae, D. pseudoobscura, D. bipectinata, D. elegans, D. rhopaloa, Sgs3b* in *D. ficusphila, Sgs1* in *D. ananassae, D. bipectinata, D. pseudoobscura, D. takahashii, D. suzukii, D. simulans, Sgs3x* in *D. pseudoobscura, D. eugracilis, D. suzukii, Sgs7* in *D. suzukii, D. ananassae, D. jambulina, D. bipectinata* and *Sgs8* in *D. suzukii.* We corrected gene annotations for: *Sgs1* in *D. ficusphila*, which had an intron disrupting its second exon sequence, *Sgs3e* in *D. obscura* and *D. subobscura* as they were missing the first exon and the intron, *Sgs3x* in *D. biarmipes and D. pseudoobscura* as the first intron was respectively missing and too long*.*

### Analysis of premature stop codons

For *D. rhopaloa Sgs1, D. ficusphila Sgs1* and *D. biarmipes Sgs3x*, premature stop codons were identified in the reference genome sequences. To examine whether they could be due to misassembly, we first BLASTed the raw reads of the respective genome sequence projects to the regions of interest and identified possible sequence corrections. Raw reads were then mapped to the coding region of interest using minimap2 (v.2.17-r941) [58] with -x map-ont parameter for nanopore reads (SRR13070618, SRR13070620) and -x splice:hq for Pacbio reads (SRR8032920). For species for which insertions were added in the corrected sequence (*D. rhopaloa, D. biarmipes*), reads were mapped to the corrected sequence whereas for *D. ficusphila* (where the sequence was corrected by removing a ‘C’ from a 6-bp stretch of C) reads were mapped to the published genome sequence. SAM files were converted to BAM file using samtools (v.1.6) and visualized in IGV (v.2.16.0) [59].

### Figure preparation

Figures were prepared using the online tool Weblogo (version 2.8.2 (2005-09-08)) (https://weblogo.berkeley.edu/logo.cgi) [60] (Fig. 1A, S11), Geneious Prime (version 2019.2.3) (https://www.geneious.com/) (Fig. 1B, 8, 11, S1, S3, S6–7, S12–15), R version 4.1.2 (2021-11-01) (https://www.r-project.org) (Figs. 2, 4, 5, 6, 7, 10, S4–5, S8, S10), IGV (v.2.16.0) (Fig. S2) and Inkscape 1.2.1 (2022-07-14 version) (https://inkscape.org/) for all figures.

### Protein alignments and their Weblogo graphical representation

Protein alignments were done using MUSCLE (3.8.425) [57] with default settings, implemented in Geneious Prime (version 2019.2.3) (https://www.geneious.com/) with the full protein sequences. Regions with at least 30% of identity were extracted and used as input sequences for the online tool Weblogo (version 2.8.2 (2005-09-08)) (https://weblogo.berkeley.edu/logo.cgi) [60] to generate sequence logos. For Fig. 1A, *Sgs3e* from *D. ananassae* and *D. bipectinata* were excluded from the alignments given the Glycine amino acid at the phase 1 intron position for *D. bipectinata* and three successive Valine amino acids in the first exon and at phase 1 intron position for *D. ananassae. Sgs3bshort* was included with *Sgs7/Sgs8* sequences and *Sgs3dshort* with *Sgs3* sequences.

### Phylogenetic trees

For *Sgs3, Sgs3x* and *Sgs1* orthologs, the aligned region containing the repeats was removed. Maximum Likelihood (ML) protein trees were then computed using PhyML (version 3.3.20180621) with default settings [61]. Bootstrap support was computed with 100 replications. Phylogenetic trees were drawn on R with the *read.dendrogram* function from the ‘ape’ package [62] and with ‘ggtree’ package (File S14).

### Identification and annotation of *Sgs* neighboring genes

To examine synteny around the *Sgs* genes, we searched for neighboring genes that tended to remain within the same locus near the *Sgs* genes in *D. yakuba*, *D. pseudoobscura*, *D. persimilis* and *D. willistoni* according to the Genomicus synteny browser (v30.01, https://www.genomicus.biologie.ens.fr/genomicus-metazoa-30.01/cgi-bin/search.pl) [63]. For the *Sgs3-Sgs7-Sgs8* gene cluster we selected the following genes: *rt*, *CG32086*, *CG7394*, *Mob2*, *Fuca*, *CG7512*, *Vha16*, *CG7551* and *CG12289*. For the *Sgs1* locus we selected: *CG3036*, *CG2831*, *hoe1*, *hoe2*, *mRpL24*, *betaggt-1* and *jet*. For the *Sgs3x* locus we selected: *AstA-R1*, *Ilp7*, *Parg*, *Mnt* and *Rala*. Sequences from *D. melanogaster* were used as BLAST queries as above to identify their homologues in other Drosophila species. When available, the NCBI gene annotations (Table S1) were collected. When no gene annotation was available or when the annotations were partial, we aligned DNA or protein sequences by using MUSCLE (see above) with global and free end gaps alignment to help in the manual annotations of the genes (Table S3 and S4). For *D. suzukii,* genes were annotated by comparison with the gene annotations of the genome of the closely related species *D. biarmipes*. *Ng* genes were found by BLAST using *D. melanogaster CG33500, CG33272, CG33270, CG43390, CG43391* amino-acid sequences as queries and by screening regions of interest. They were manually annotated based on start and stop codons as they are intronless. We note that other genes not found by our BLAST searches are also annotated as ‘protein new glue’ in several Drosophila genomes. We did not consider them in this study. Their phylogenetic relationship with the new *ng* genes we identified remains to be investigated.

### Visualization of genomic region alignments with Easyfig and Genoplot

We used Easyfig (version 2.2.2) (https://mjsull.github.io/Easyfig/) [64] to compare *Sgs* genomic regions between species. As input for the EasyFig software, we used annotated genomic regions. EasyFig performs blastn searches on a one-by-one species comparison, starting from the first species, so that each sequence is used as a blast query for the next species in the list. We used the following BLAST parameters: Min. length (minimum length of blast hits to be drawn) = 0, Max. e Value (Maximum expected value of blast hits to be drawn) = 0.001, Min identity Value (Minimum identity value of blast hits to be drawn) = 0. We collected the Easyfig output files (.out) and processed them through the Genoplot package [65] (R version 4.1.2 (2021-11-01) (https://www.r-project.org.)) to generate figures of sequence alignments. Genbank files were read with the function read.dna_seg from the Genoplot package. Colors and text on the figures generated with Genoplot were added with Inkscape 1.2.1 (9c6d41e410, 2022-07-14).

### Dotplots

Dotplot drawing program in Geneious Prime (version 2019.2.3) (https://www.geneious.com/) was used to compare two genomic regions. We used the following parameters: High Sensitivity/Slow: sliding window, Score Matrix: Probabilistic: Weighted Ambiguous Matches, window size: 50, threshold: 100.

### Repeats analysis

We examined genomic regions of 129 kb with the *Sgs* genes of interest being in the middle of the region. On Geneious Prime (version 2019.2.3) (https://www.geneious.com/), we used the FindRepeats plugin to annotate regions that are repeated at least once within a given sequence. We used the following criteria: minimum repeat length: 20 bp, maximum mismatches: 5. The repeat annotations were then transformed into bar plots representing the number of base pairs harboring repeats in adjacent windows of 1 kb using a custom-made R script (File S9).

### Protein motif scanning

We used ScanProsite tool [66] (Release 2022_04 of 12-Oct-2022) (https://prosite.expasy.org/scanprosite/) to search for the protein motifs obtained from Fig. 1 against the protein sequence database given by ScanProsite. We chose ‘Option 2 - Submit MOTIFS to scan them against a PROTEIN sequence database’ and used the default settings.

### Supplementary Information

**Additional file 1.**

## Data Availability

The genome sequence assembly and annotation data used in this study can be retrieved at NCBI with the hyperlinks indicated in Table S1. Raw data, alignments and scripts are available as supplementary files associated with this article GenBank accession number (*D. takahashii* partial *Sgs1* sequence): OP857324.

## Abbreviations

*Sgs*: Salivary gland secretion genes
*Ng*: New glue genes

## References

[1] Demuth JP, Hahn MW (2009). The life and death of gene families. Bioessays..

[2] Kondrashov FA (2012). Gene duplication as a mechanism of genomic adaptation to a changing environment. Proc R Soc B Biol Sci..

[3] Courtier-Orgogozo V, Arnoult L, Prigent SR, Wiltgen S, Martin A. Gephebase, a Database of Genotype-Phenotype Relationships for natural and domesticated variation in Eukaryotes. BioRxiv. 2019;618371. 10.1093/nar/gkz796 PMC6943045 31544935

[4] Albalat R, Cañestro C (2016). Evolution by gene loss. Nat Rev Genet..

[5] Lye ZN, Purugganan MD (2019). Copy number variation in domestication. Trends Plant Sci..

[6] Redon R, Ishikawa S, Fitch KR, Feuk L, Perry GH, Andrews TD (2006). Global variation in copy number in the human genome. Nature..

[7] Hayden S, Bekaert M, Crider TA, Mariani S, Murphy WJ, Teeling EC (2010). Ecological adaptation determines functional mammalian olfactory subgenomes. Genome Res..

[8] Birchler JA, Veitia RA (2007). The gene balance hypothesis: from classical genetics to modern genomics. Plant Cell..

[9] Carroll SB (2008). Evo-devo and an expanding evolutionary synthesis: a genetic theory of morphological evolution. Cell..

[10] Defoort J, Van de Peer Y, Carretero-Paulet L (2019). The evolution of gene duplicates in angiosperms and the impact of protein–protein interactions and the mechanism of duplication. Genome Biol Evol..

[11] Hastings PJ, Lupski JR, Rosenberg SM, Ira G (2009). Mechanisms of change in gene copy number. Nat Rev Genet..

[12] Feschotte C, Pritham EJ (2007). DNA transposons and the evolution of eukaryotic genomes. Annu Rev Genet..

[13] Xie KT, Wang G, Thompson AC, Wucherpfennig JI, Reimchen TE, MacColl AD (2019). DNA fragility in the parallel evolution of pelvic reduction in stickleback fish. Science..

[14] El-Mabrouk N (2021). Predicting the Evolution of Syntenies—An Algorithmic Review. Algorithms..

[15] Walker EL, Robbins TP, Bureau TE, Kermicle J, Dellaporta SL (1995). Transposon-mediated chromosomal rearrangements and gene duplications in the formation of the maize R-r complex. EMBO J..

[16] Menardo F, Praz CR, Wicker T, Keller B (2017). Rapid turnover of effectors in grass powdery mildew (Blumeria graminis). BMC Evol Biol..

[17] Schmidt JM, Good RT, Appleton B, Sherrard J, Raymant GC, Bogwitz MR (2010). Copy number variation and transposable elements feature in recent, ongoing adaptation at the Cyp6g1 locus. PLoS Genet..

[18] Pajic P, Pavlidis P, Dean K, Neznanova L, Romano R-A, Garneau D (2019). Independent amylase gene copy number bursts correlate with dietary preferences in mammals. eLife..

[19] Ishikawa A, Kabeya N, Ikeya K, Kakioka R, Cech JN, Osada N (2019). A key metabolic gene for recurrent freshwater colonization and radiation in fishes. Science..

[20] Denton JF, Lugo-Martinez J, Tucker AE, Schrider DR, Warren WC, Hahn MW (2014). Extensive error in the number of genes inferred from draft genome assemblies. PLoS Comput Biol..

[21] Han MV, Thomas GW, Lugo-Martinez J, Hahn MW (2013). Estimating gene gain and loss rates in the presence of error in genome assembly and annotation using CAFE 3. Mol Biol Evol..

[22] Hahn MW, Han MV, Han S-G (2007). Gene family evolution across 12 Drosophila genomes. PLoS Genet..

[23] Borne F, Kulathinal RJ, Courtier-Orgogozo V (2021). Glue genes are subjected to diverse selective forces during Drosophila development. Genome Biol Evol..

[24] Monier M, Courtier-Orgogozo V (2022). Drosophila Glue: A Promising Model for Bioadhesion. Insects..

[25] Da Lage J-L, Thomas GW, Bonneau M, Courtier-Orgogozo V (2019). Evolution of salivary glue genes in Drosophila species. BMC Evol Biol..

[26] Syed ZA, Härd T, Uv A, van Dijk-Härd IF (2008). A potential role for Drosophila mucins in development and physiology. PLoS One..

[27] Korayem AM, Fabbri M, Takahashi K, Scherfer C, Lindgren M, Schmidt O (2004). A Drosophila salivary gland mucin is also expressed in immune tissues: evidence for a function in coagulation and the entrapment of bacteria. Insect Biochem Mol Biol..

[28] Kim BY, Wang JR, Miller DE, Barmina O, Delaney E, Thompson A (2021). Highly contiguous assemblies of 101 drosophilid genomes. Elife..

[29] Altschul S (1997). Gapped BLAST and PSI-BLAST: a new generation of protein database search programs. Nucleic Acids Res..

[30] Farkaš R (2016). The complex secretions of the salivary glands of Drosophila melanogaster, a model system. Extracellular composite matrices in Arthropods.

[31] Chiu JC, Jiang X, Zhao L, Hamm CA, Cridland JM, Saelao P (2013). Genome of Drosophila suzukii, the spotted wing drosophila. G3 Genes Genomes Genet..

[32] Paris M, Boyer R, Jaenichen R, Wolf J, Karageorgi M, Green J (2020). Near-chromosome level genome assembly of the fruit pest Drosophila suzukii using long-read sequencing. Sci Rep..

[33] Suvorov A, Kim BY, Wang J, Armstrong EE, Peede D, D’agostino ER (2022). Widespread introgression across a phylogeny of 155 Drosophila genomes. Curr Biol..

[34] Kumar S, Stecher G, Suleski M, Hedges SB (2017). TimeTree: a resource for timelines, timetrees, and divergence times. Mol Biol Evol..

[35] Furia M, Digilio FA, Artiaco D, Giordano E, Polito LC (1990). A new gene nested within the dunce genetic unit of *Drosophila melanogaster*. Nucleic Acids Res..

[36] Furia M, D’Avino PP, Crispi S, Artiaco D, Polito LC (1993). Dense Cluster of Genes is Located at the Ecdysone-regulated 3C Puff of Drosphila melanogaster. J Mol Biol..

[37] Tokareva O, Jacobsen M, Buehler M, Wong J, Kaplan DL (2014). Structure–function–property–design interplay in biopolymers: Spider silk. Acta Biomater..

[38] Martin CH, Meyerowitz EM (1986). Characterization of the boundaries between adjacent rapidly and slowly evolving genomic regions in Drosophila. Proc Natl Acad Sci..

[39] Lehmann M, Wattler F, Korge G (1997). Two new regulatory elements controlling the Drosophila Sgs-3 gene are potential ecdysone receptor and fork head binding sites. Mech Dev..

[40] Giangrande A, Mettling C, Richards G (1987). Sps-3 transcript levels are determined by multiple remote sequence elements. EMBO J..

[41] Roth GE, Wattler S, Bornschein H, Lehmann M, Korge G (1999). Structure and regulation of the salivary gland secretion protein gene Sgs-1 of *Drosophila melanogaster*. Genetics..

[42] Biyasheva A, Do T-V, Lu Y, Vaskova M, Andres AJ (2001). Glue secretion in the Drosophila salivary gland: a model for steroid-regulated exocytosis. Dev Biol..

[43] Shore EM, Guild GM (1987). Closely linked DNA elements control the expression of the Sgs-5 glue protein gene in Drosophila. Genes Dev..

[44] Hofmann A, Garfinkel MD, Meyerowitz EM (1991). cis-acting sequences required for expression of the divergently transcribed *Drosophila melanogaster* Sgs-7 and Sgs-8 glue protein genes. Mol Cell Biol..

[45] Benton R (2015). Multigene family evolution: perspectives from insect chemoreceptors. Trends Ecol Evol..

[46] Li T-R, White KP (2003). Tissue-specific gene expression and ecdysone-regulated genomic networks in Drosophila. Dev Cell..

[47] Liu Y, Lehmann M (2008). Genes and biological processes controlled by the Drosophila FOXA orthologue Fork head. Insect Mol Biol..

[48] Ryuda M, Shimada K, Koyanagi R, Azumi K, Tanimura T, Hayakawa Y (2008). Analysis of hunger-driven gene expression in the Drosophila melanogaster larval central nervous system. Zoolog Sci..

[49] D’Avino PP, Crispi S, Polito LC, Furia M (1995). The role of the BR-C locus on the expression of genes located at the ecdysone-regulated 3C puff of Drosophila melanogaster. Mech Dev..

[50] Casas-Vila N, Bluhm A, Sayols S, Dinges N, Dejung M, Altenhein T (2017). The developmental proteome of Drosophila melanogaster. Genome Res..

[51] Crispi S, Giordano E, D’Avino PP, Peluso I, Furia M (2001). Functional analysis of regulatory elements controlling the expression of the ecdysone-regulated Drosophila ng-1 gene. Mech Dev..

[52] Crispi S, Giordano E, D’Avino PP, Furia M (1998). Cross-talking among Drosophila nuclear receptors at the promiscuous response element of the ng-1 and ng-2 intermolt genes. J Mol Biol..

[53] Lewis RV (2006). Spider silk: ancient ideas for new biomaterials. Chem Rev..

[54] Casewell NR, Jackson TN, Laustsen AH, Sunagar K (2020). Causes and consequences of snake venom variation. Trends Pharmacol Sci..

[55] Baker RH, Corvelo A, Hayashi CY (2022). Rapid molecular diversification and homogenization of clustered major ampullate silk genes in Argiope garden spiders. PLoS Genet..

[56] Dowell NL, Giorgianni MW, Kassner VA, Selegue JE, Sanchez EE, Carroll SB (2016). The deep origin and recent loss of venom toxin genes in rattlesnakes. Curr Biol..

[57] Edgar RC (2004). MUSCLE: multiple sequence alignment with high accuracy and high throughput. Nucleic Acids Res..

[58] Li H (2018). Minimap2: pairwise alignment for nucleotide sequences. Bioinformatics..

[59] Thorvaldsdóttir H, Robinson JT, Mesirov JP (2013). Integrative Genomics Viewer (IGV): high-performance genomics data visualization and exploration. Brief Bioinform..

[60] Crooks GE, Hon G, Chandonia J-M, Brenner SE (2004). WebLogo: a sequence logo generator. Genome Res..

[61] Guindon S, Dufayard J-F, Lefort V, Anisimova M, Hordijk W, Gascuel O (2010). New algorithms and methods to estimate maximum-likelihood phylogenies: assessing the performance of PhyML 3.0. Syst Biol..

[62] Paradis E, Claude J, Strimmer K (2004). APE: analyses of phylogenetics and evolution in R language. Bioinformatics..

[63] Nguyen NTT, Vincens P, Roest Crollius H, Louis A (2018). Genomicus 2018: karyotype evolutionary trees and on-the-fly synteny computing. Nucleic Acids Res..

[64] Sullivan MJ, Petty NK, Beatson SA (2011). Easyfig: a genome comparison visualizer. Bioinformatics..

[65] Guy L, Roat Kultima J, Andersson SG (2010). genoPlotR: comparative gene and genome visualization in R. Bioinformatics..

[66] De Castro E, Sigrist CJ, Gattiker A, Bulliard V, Langendijk-Genevaux PS, Gasteiger E (2006). ScanProsite: detection of PROSITE signature matches and ProRule-associated functional and structural residues in proteins. Nucleic Acids Res..

