# Supplementary material for: Higher evolutionary dynamics of gene copy number for Drosophila glue genes located near short repeat sequences
Source: BMC Ecol Evol. 2024 Feb 2;24:18. doi: 10.1186/s12862-023-02178-y (PMC10835880; doi:10.1186/s12862-023-02178-y)
Supplement: Supplementary file 1 — Additional file 1. [file 12862_2023_2178_MOESM1_ESM.zip › Supplementary/manuscript_supp.pdf]

Higher evolutionary dynamics of gene copy number for Drosophila glue genes located near short repeat sequences

Supplementary Figures

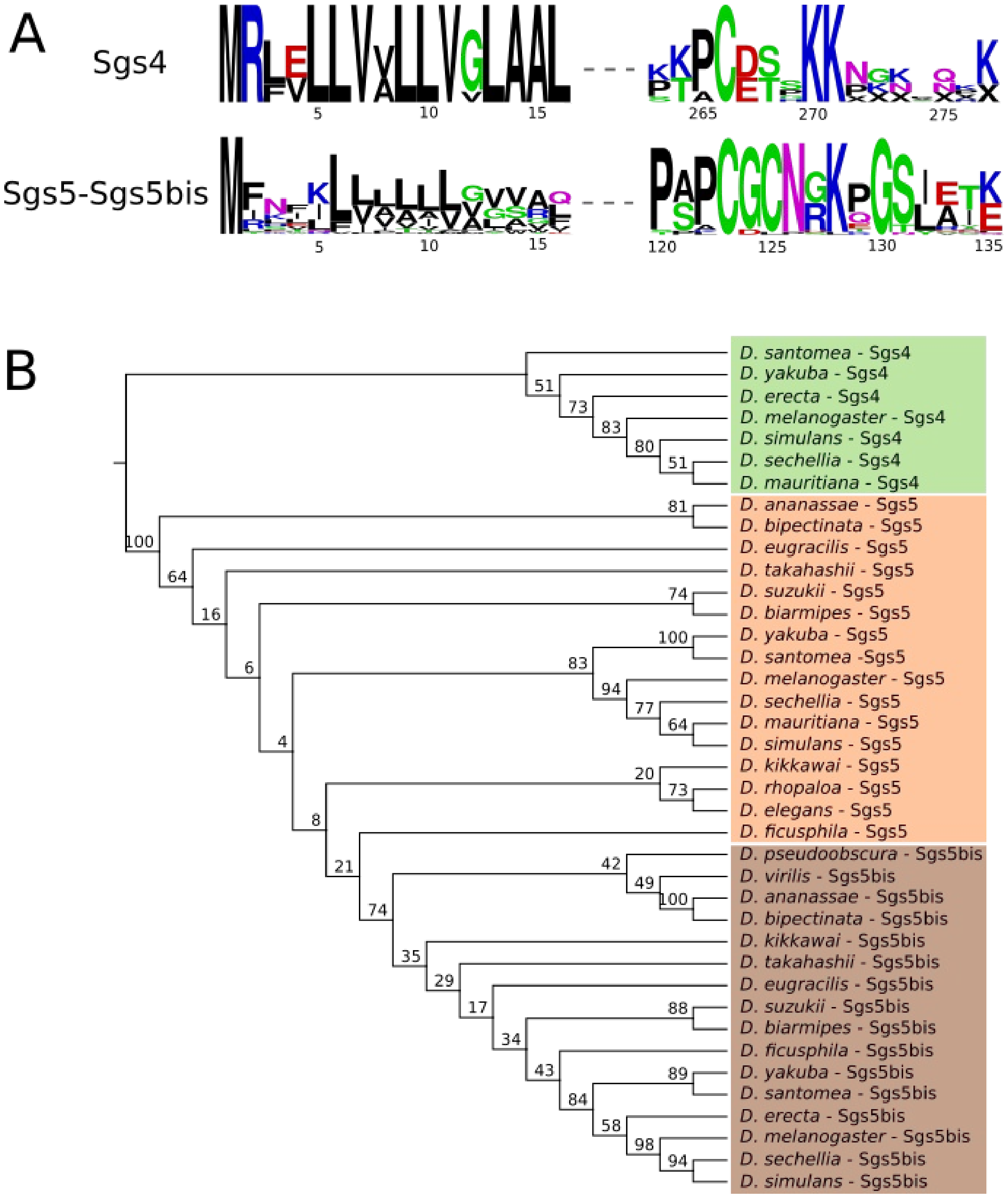

Fig. S1. Overview of the Sgs4, Sgs5 and Sgs5bis proteins in Drosophila. (A) Conserved amino acid motifs in Sgs4 and Sgs5-Sgs5bis proteins. Same legend as Fig. 1A. (B) Maximum likelihood (ML) tree of aligned, full Sgs4, Sgs5 and Sgs5bis amino acid sequences. Numbers along branches are bootstrap values. The tree was rooted between the Sgs4 cluster and the Sgs5-Sgs5bis cluster.

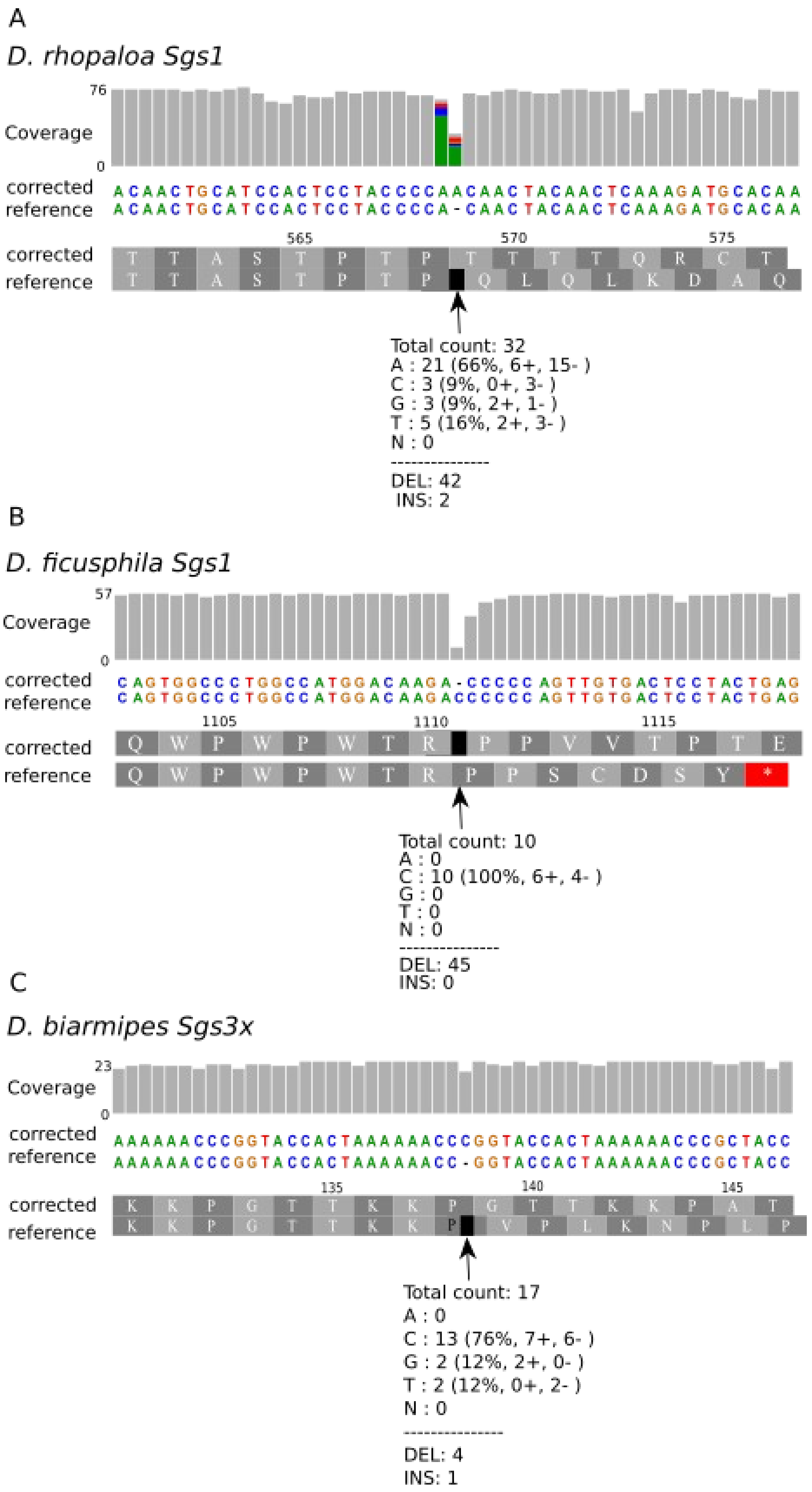

Fig. S2. Analysis of premature stop codons in three Sgs genes. Raw reads from respective full genome sequencing projects suggest that (A) D. rhopaloa Sgs1 nucleotide reference sequence should be corrected by adding an ‘A’ nucleotide, (B) D. ficusphila Sgs1 nucleotide sequence by deleting a ‘C’ and (C) D. biarmipes Sgs3x nucleotide sequence by adding a 'C'. In each panel, top gray bars represent the coverage of raw reads mapped to the corrected

(A,C) or reference (B) sequence. Reference and corrected sequences are indicated below. The distribution of nucleotides and indels at the site of interest is presented below. DEL: deletion; INS: insertion.

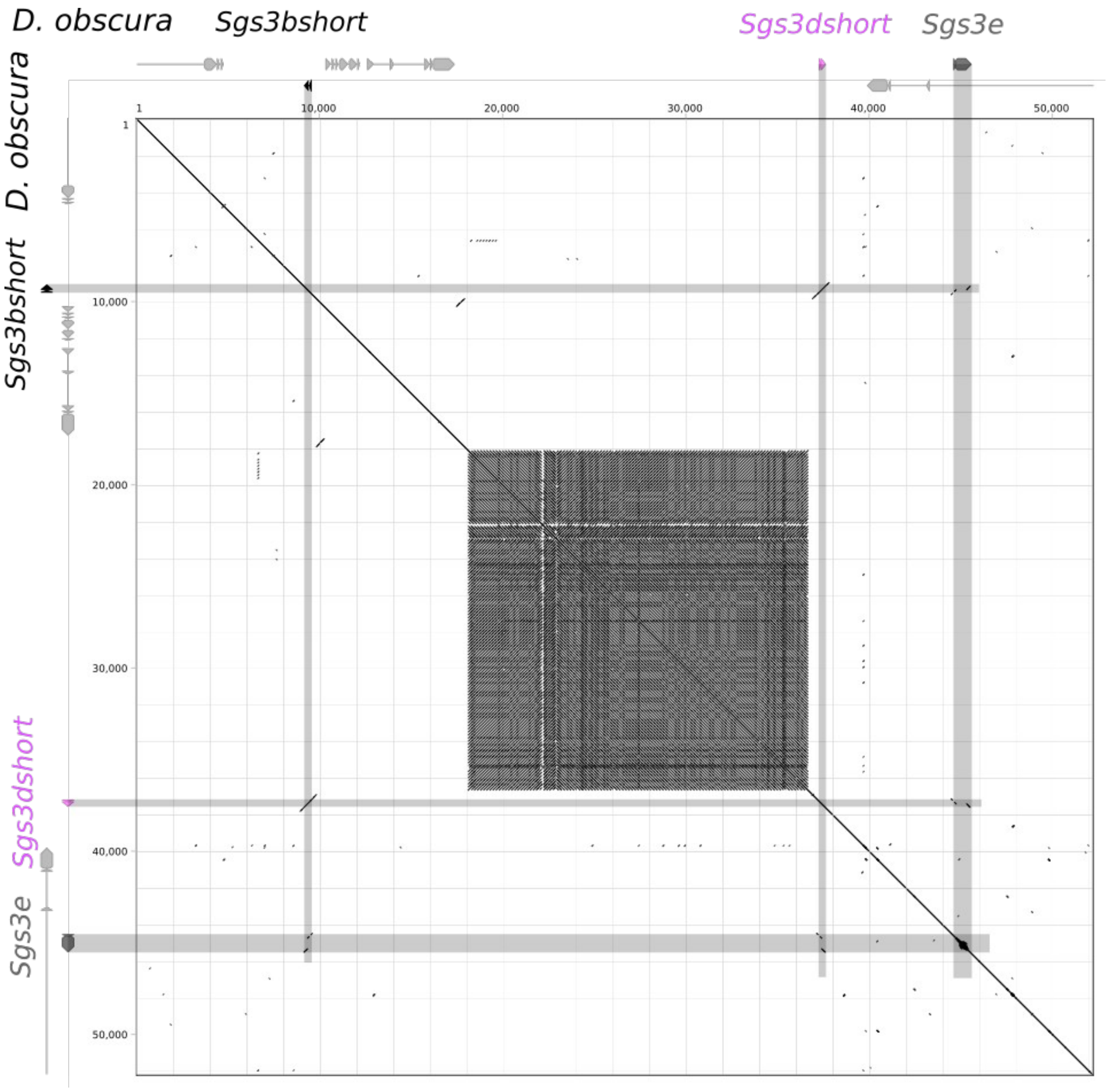

Fig. S3. Dot plot comparison of *D. obscura Sgs3* genomic region with itself. Black, light pink and dark grey arrows represent, respectively, *Sgs3bshort*, *Sgs3dshort* and *Sgs3e*. Light grey arrows represent neighboring genes. Numbers indicate nucleotide positions in bp.

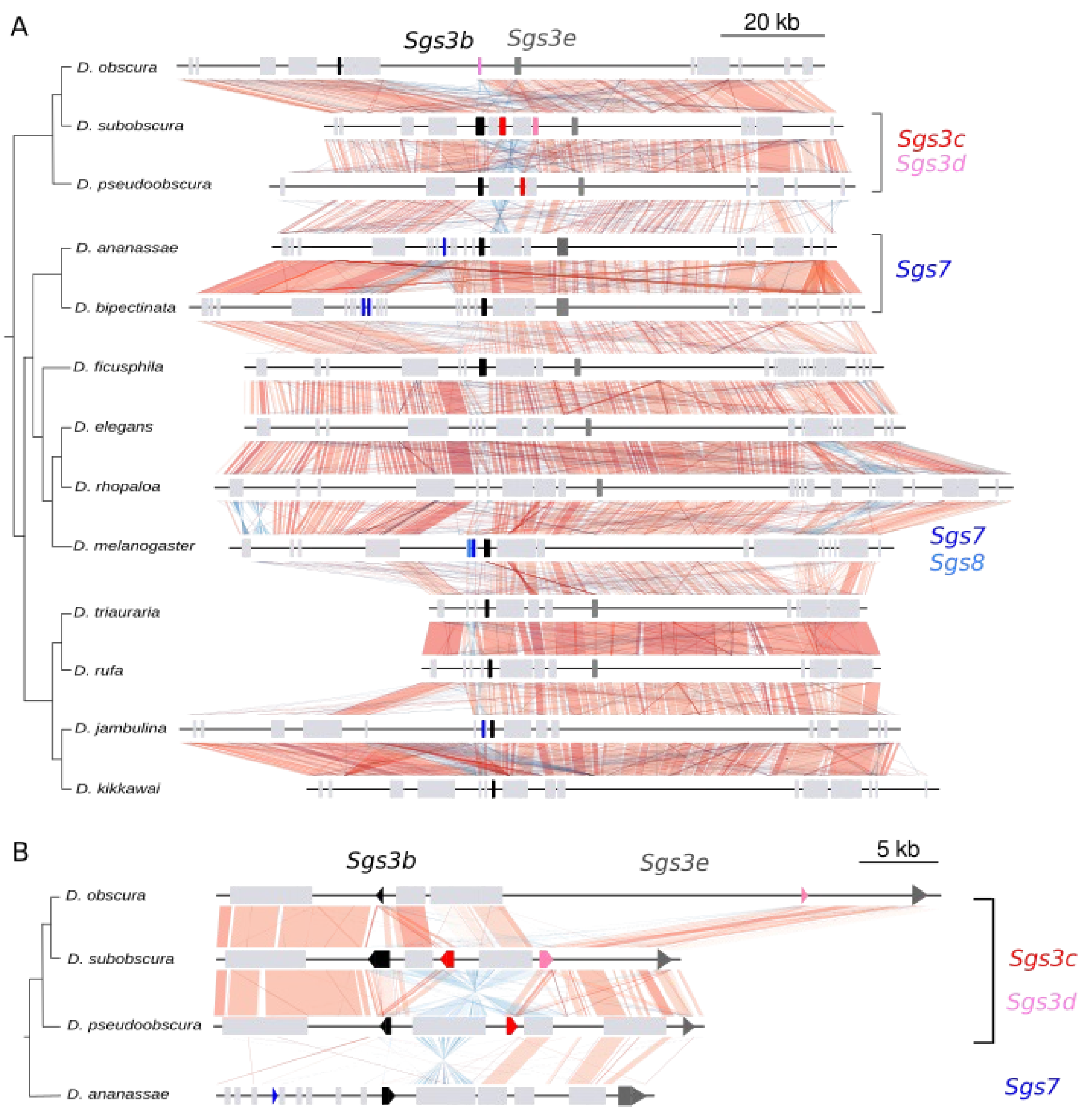

Fig. S4. Comparison of the *Sgs3-7-8* gene region between Drosophila species. (A) Entire locus comprising *Sgs3*, *Sgs7* and *Sgs8* genes. (B) Magnification of the *Sgs3b-Sgs3e* region. Same legend as in Fig. 5. *Sgs7* copies are in dark blue, *Sgs8* in light blue. *Sgs3b, Sgs3c, Sgs3d, Sgs3e* copies are respectively represented in black, red, light pink, dark gray. *Sgs3bshort* and *Sgs3dshort* are respectively in black and light pink and are only found in *D. obscura*. *Mob2* gene has been removed from this representation for the sake of clarity because it is superposed with *Sgs3e*.

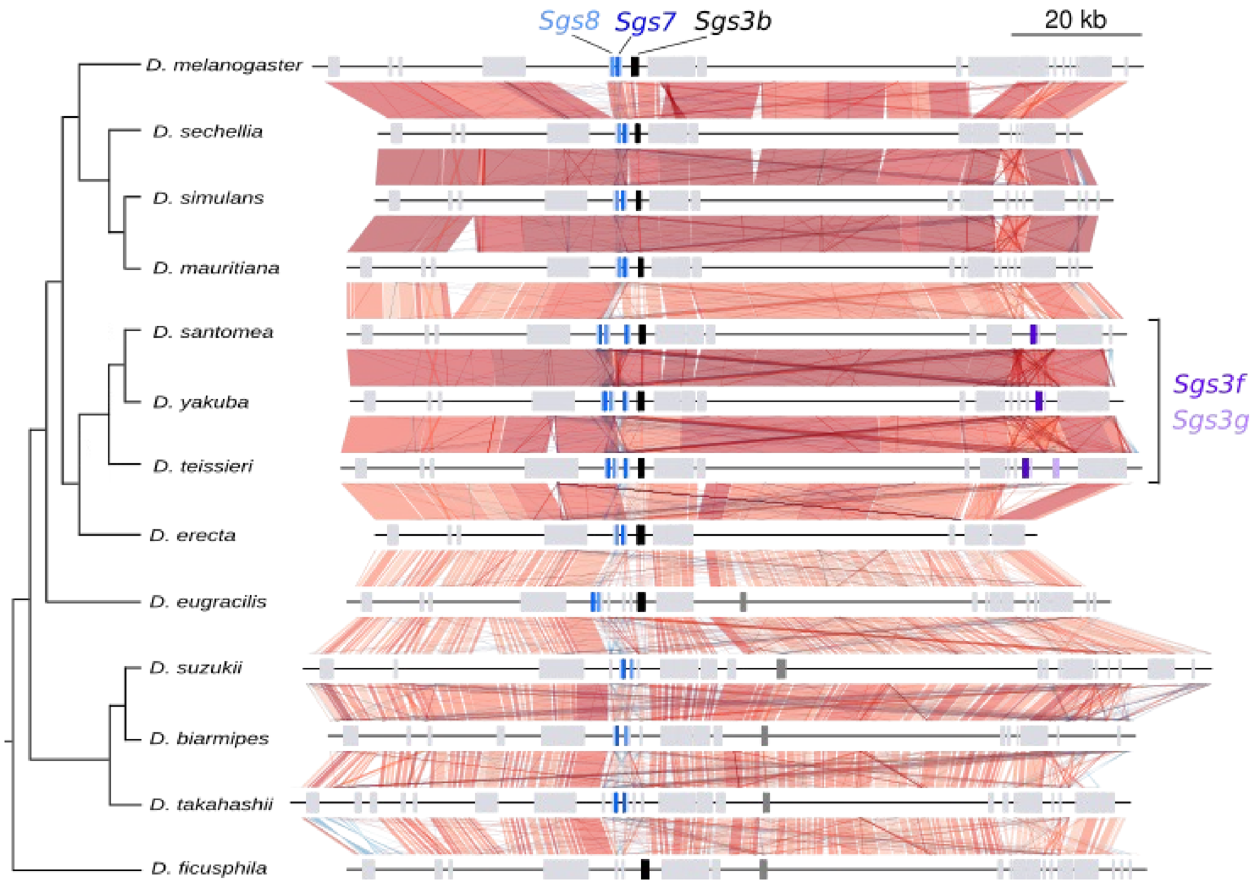

Fig. S5. Comparison of the *Sgs3-7-8* gene region between Drosophila species. Same legend as in Fig. 5. *Sgs7* copies are in dark blue, *Sgs8* in light blue. *Sgs3b*, *Sgs3e*, *Sgs3f* and *Sgs3g* copies are represented in black, dark grey, dark and light purple, respectively.

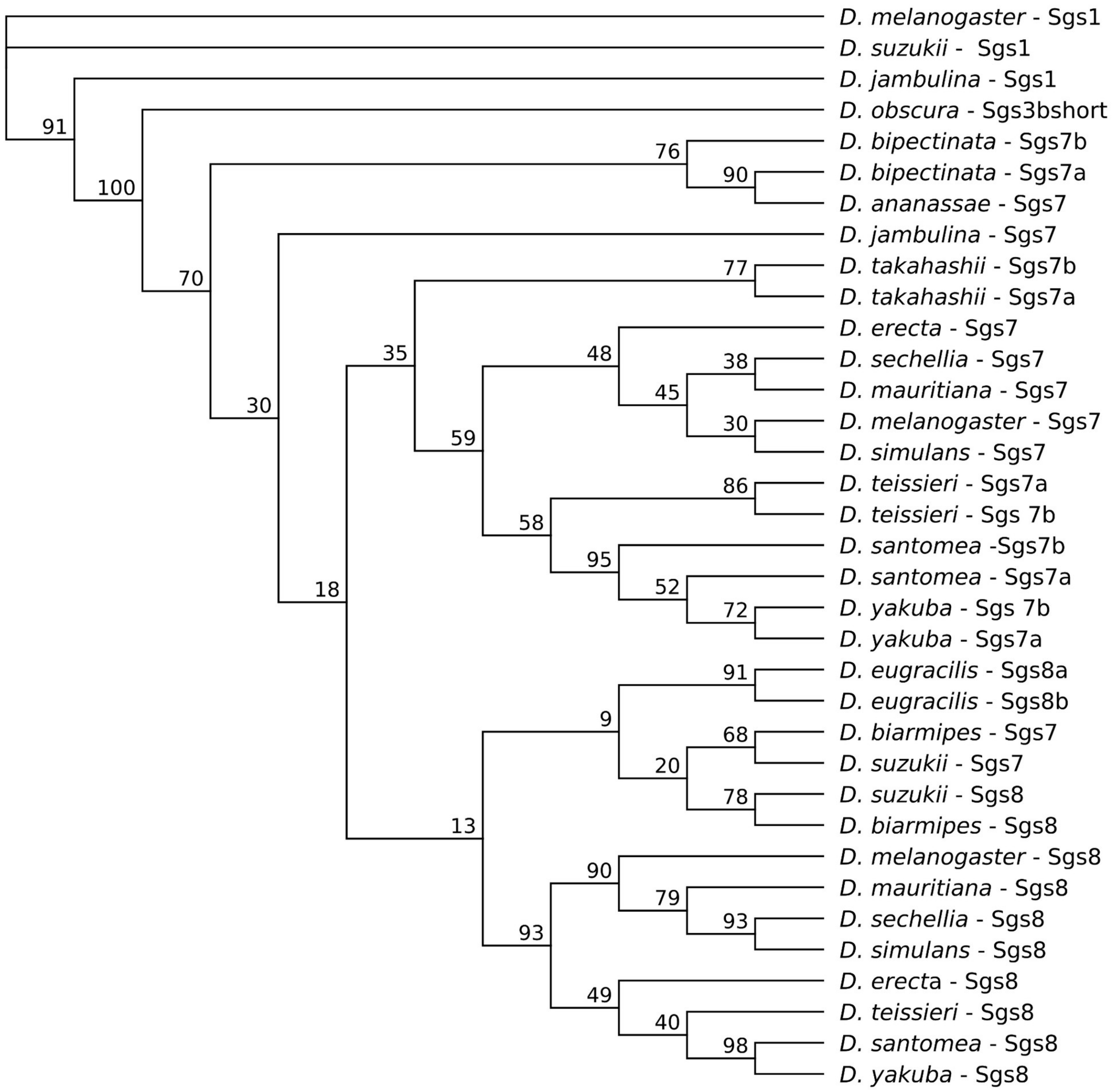

Fig. S6. Maximum likelihood (ML) unrooted tree (amino acid sequences) for *Sgs7* and *Sgs8* from all studied species and using a few outgroup sequences (*Sgs1* from *D. melanogaster, D. suzukii, D. jambulina and Sgs3bshort* from *D. obscura).* Numbers along branches indicate bootstrap values.

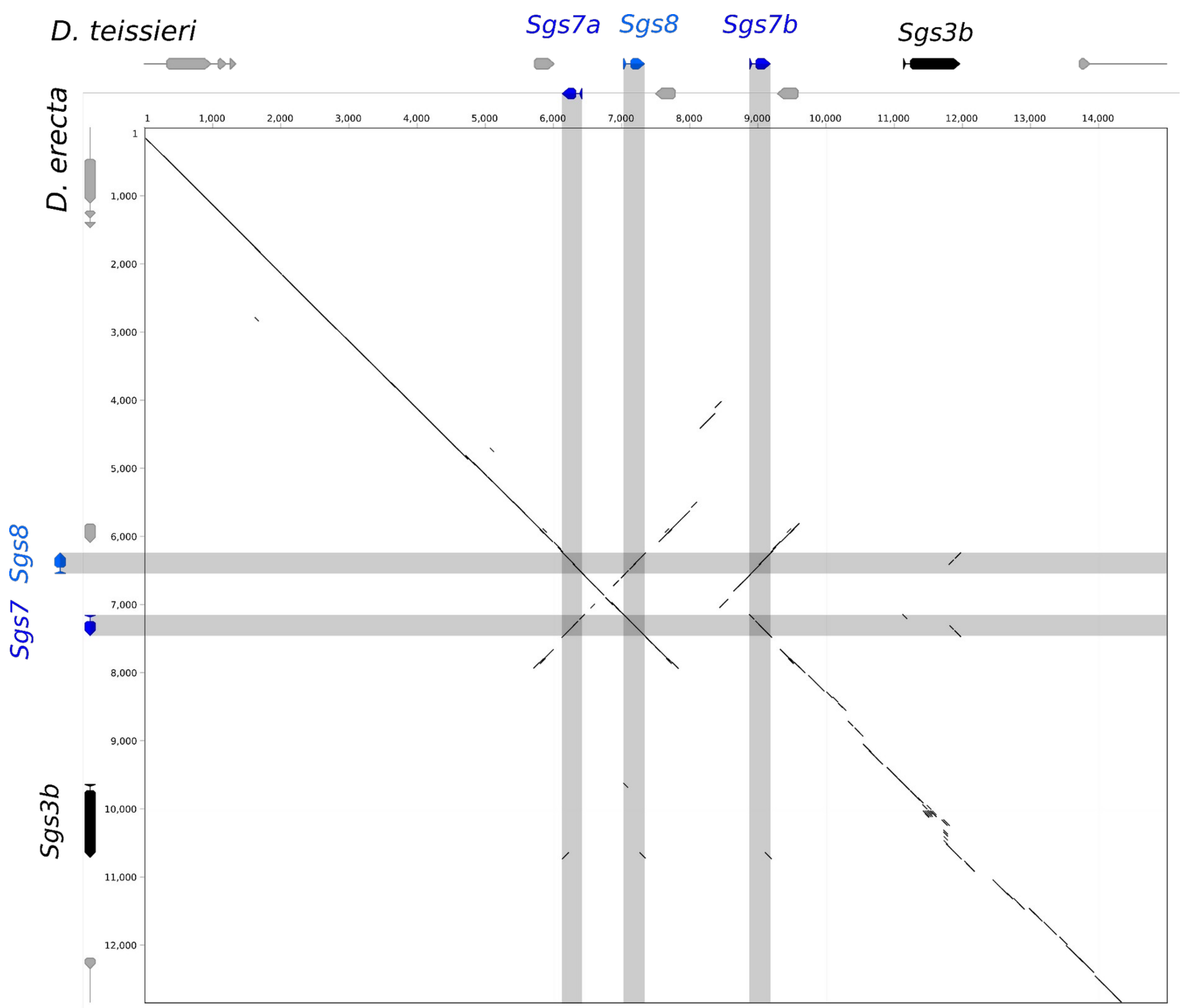

Fig. S7. Dot plot comparison of *Sgs3-7-8* genomic regions from *D. erecta* and *D. teissieri*. Black diagonal lines indicate matching genomic regions. Black, dark blue and light blue arrows represent, respectively, *Sgs3b*, *Sgs7* and *Sgs8* orthologs. Same legend as Fig. S1.

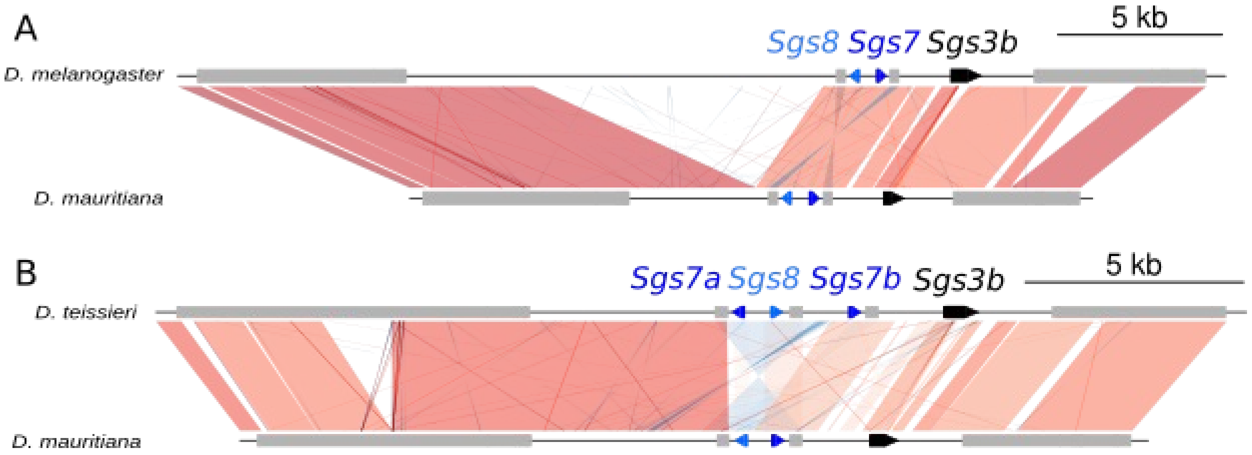

Fig. S8. Comparison of the *Sgs3-7-8* genomic region between *D. melanogaster, D. mauritiana* and *D. teissieri*. Same legend as in Fig. 4 (A) The orientation and position of *Sgs7, Sgs8 and Sgs3b is similar between D. melanogaster* and *D. mauritiana*. (B) The *Sgs3-7-8* genomic region experienced a gene duplication of *Sgs7* and an inversion affecting *Sgs7a* and *Sgs8* genes (light blue hourglass shape) in the lineage leading to *D. teissieri* (see also Fig. 9). One of the breakpoints of the inversion is a *ng* gene (gray box).

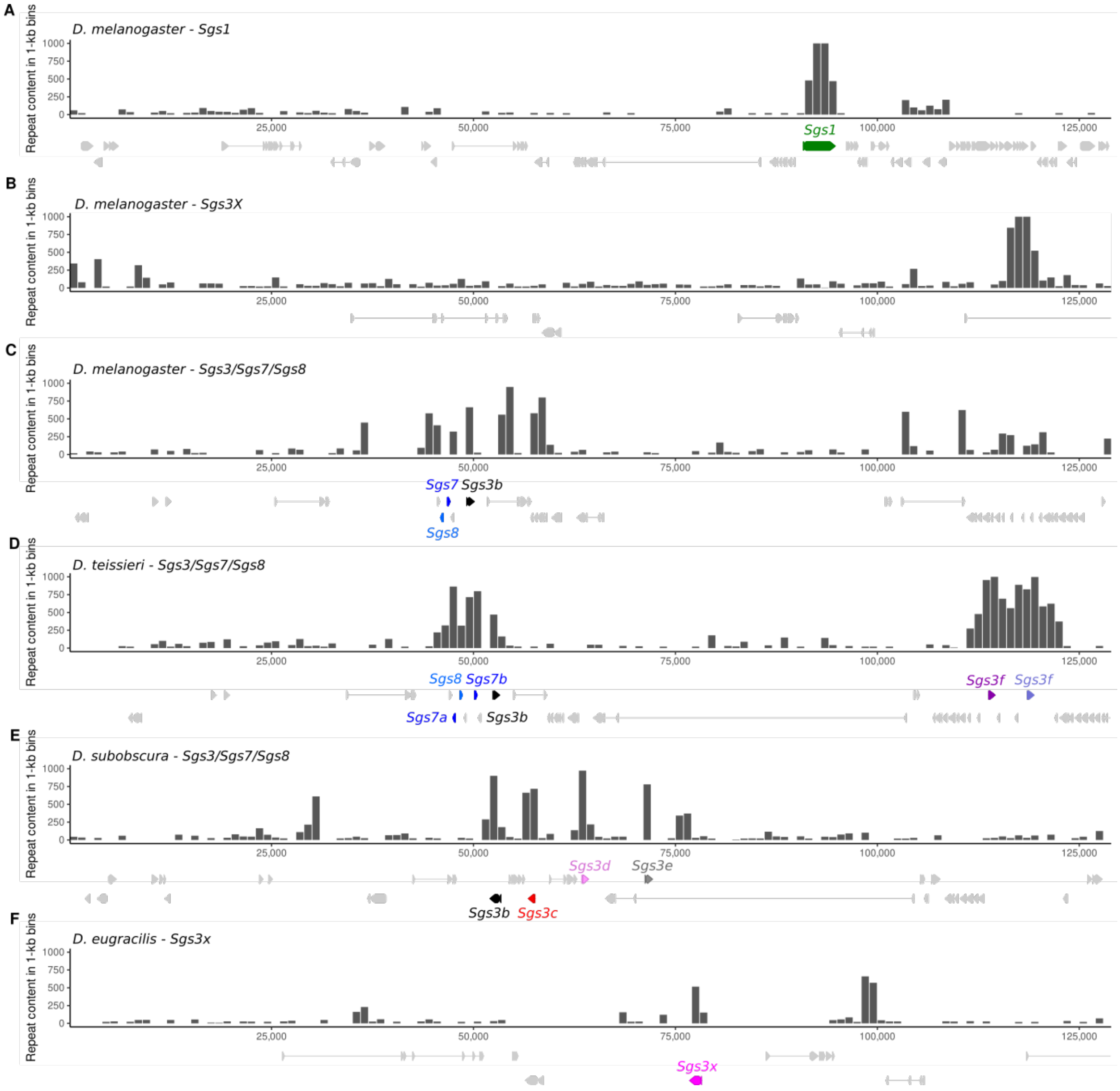

Fig. S9. Amount of repeats in 129-kb *Sgs* genomic sequences of several Drosophila species. Each bar represents the number of nucleotides within a 1-kb window that are annotated as repeats. The annotations of *Sgs* genes and neighboring genes are displayed with arrows. Sgs3x is absent in *D. melanogaster*. Note that internal repeats present within the coding regions of *Sgs* genes are annotated as repeats.

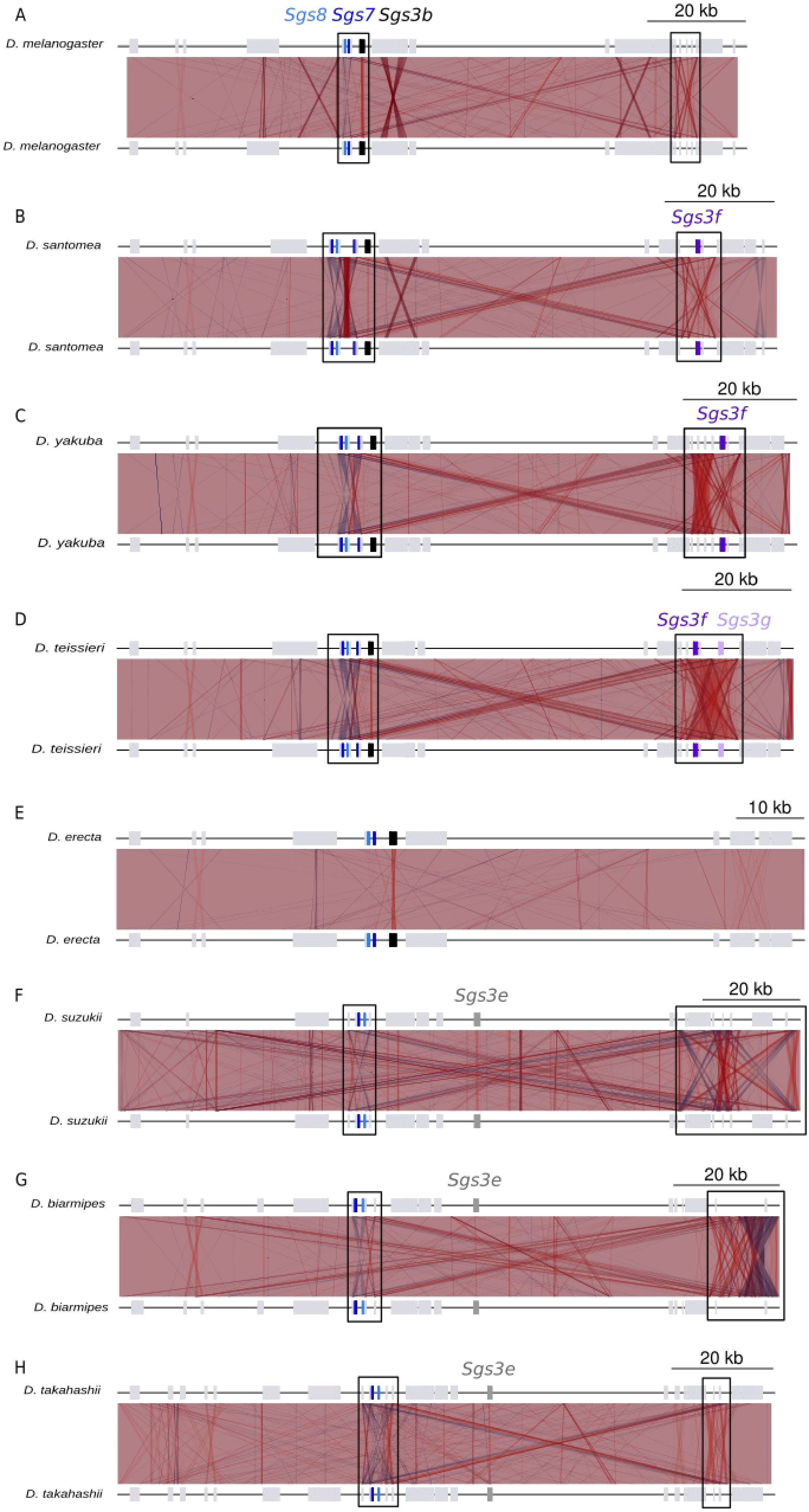

A
Sgs8 Sgs7 Sgs3b
20 kb
D. melanogaster
D. melanogaster
B
20 kb
Sgs3f
D. santomea
D. santomea
C
20 kb
Sgs3f
D. yakuba
D. yakuba
D
20 kb
Sgs3f Sgs3g
D. teissieri
D. teissieri
E
10 kb
D. erecta
D. erecta
F
Sgs3e
20 kb
D. suzukii
D. suzukii
G
Sgs3e
20 kb
D. biarmipes
D. biarmipes
H
Sgs3e
20 kb
D. takahashii
D. takahashii

Fig. S10. Comparison of the *Sgs3-7-8* genomic region from several Drosophila species with itself. Same legend as in Fig. S2. *Mob2* gene has been removed from this representation for the sake of readability because it is superposed with one of *Sgs3* copies. Black frames highlight two different genomic regions matching between each other, as we can see by the dark red lines forming a ‘cross’ pattern between the two loci. Comparison for *D. melanogaster* (A), *D. santomea* (B), *D. yakuba* (C), *D. teissieri* (D), *D. erecta* (E), *D. suzukii* (F), *D. biarmipes* (G) and *D. takahashii* (H).

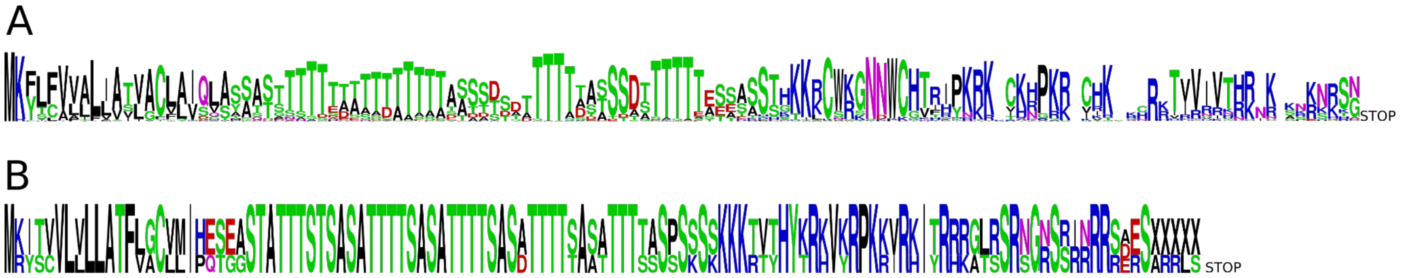

Fig. S11. Conserved amino acid sequence of ng proteins. Same legend as in Fig.1A. Numbers indicate the positions of the amino acid in *D. melanogaster CG33500* protein. (A) Conserved amino acid sequence based on an alignment of 154 ng proteins from all studied species. (B) Conserved amino acid sequence for three previously annotated *ng* genes at position 3C1 in *D. melanogaster (ng1, ng2, ng3*). We did not include previously annotated *ng4* gene because it is missing threonine-rich repeats.

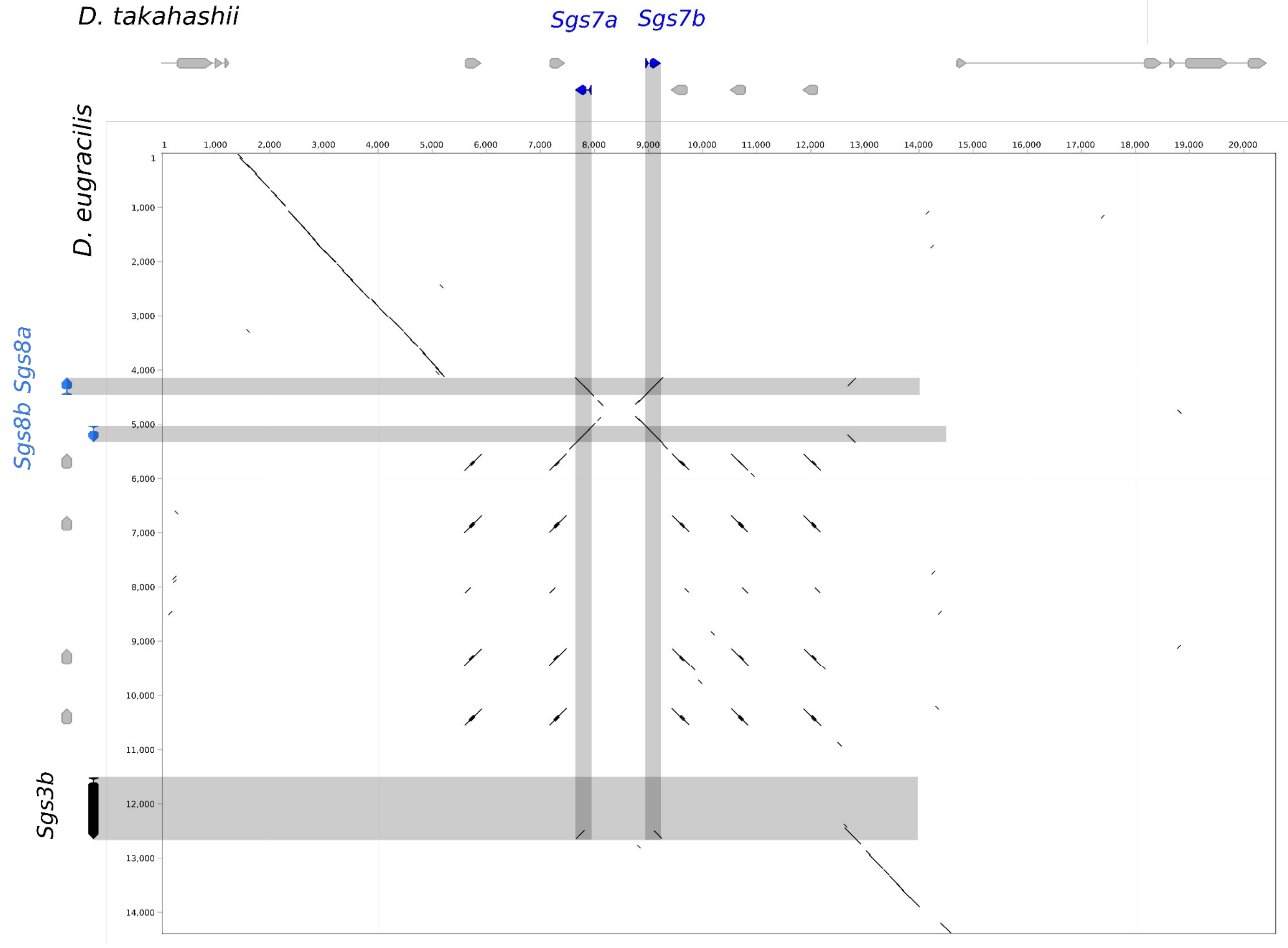

Fig S12. Dotplot comparison of *Sgs3-7-8* genomic regions from *D. eugracilis* and *D. takahashii.* Same legend as Fig. S1.

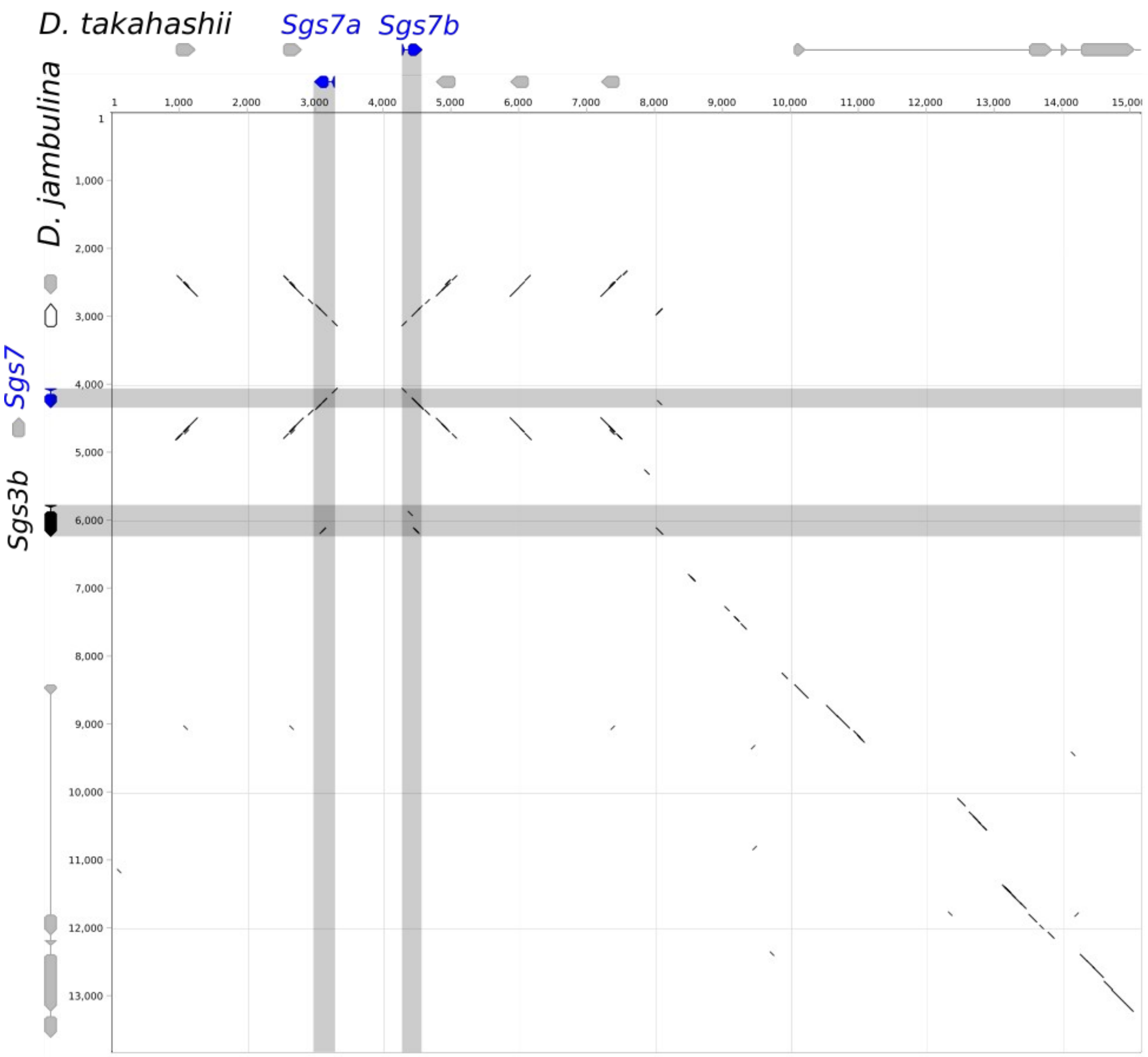

Fig S13. Dot plot comparison of *Sgs3-7-8* genomic regions from *D. takahashii* and *D. jambulina.* Same legend as Fig. S5. The white arrow indicates an *Sgs* pseudogene in *D. jambulina* which is missing the first coding exon and the start codon.

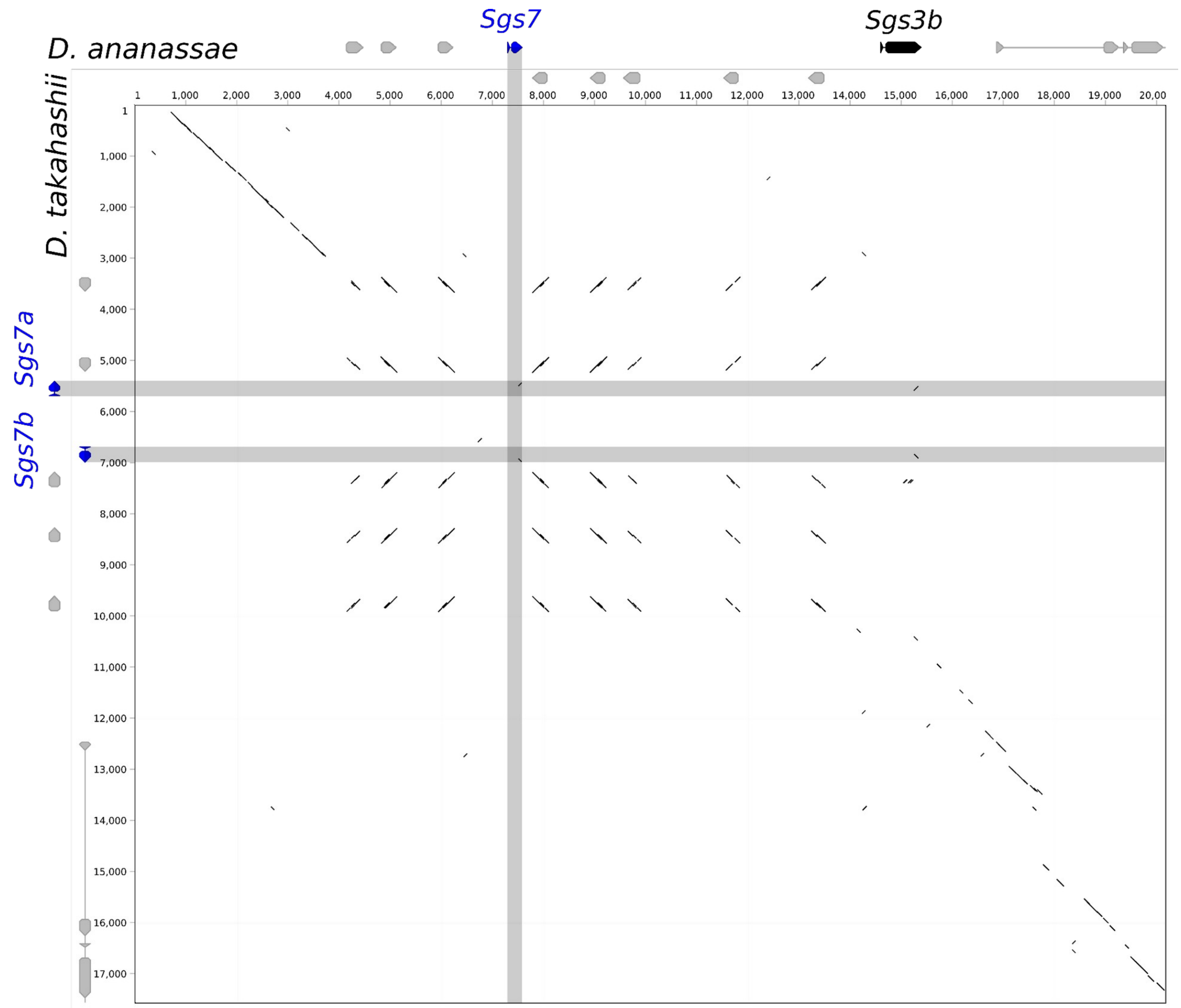

Fig S14. Dot plot comparison of *Sgs3-7-8* genomic regions from *D. ananassae* and *D. takahashii*. Same legend as Fig. S1.

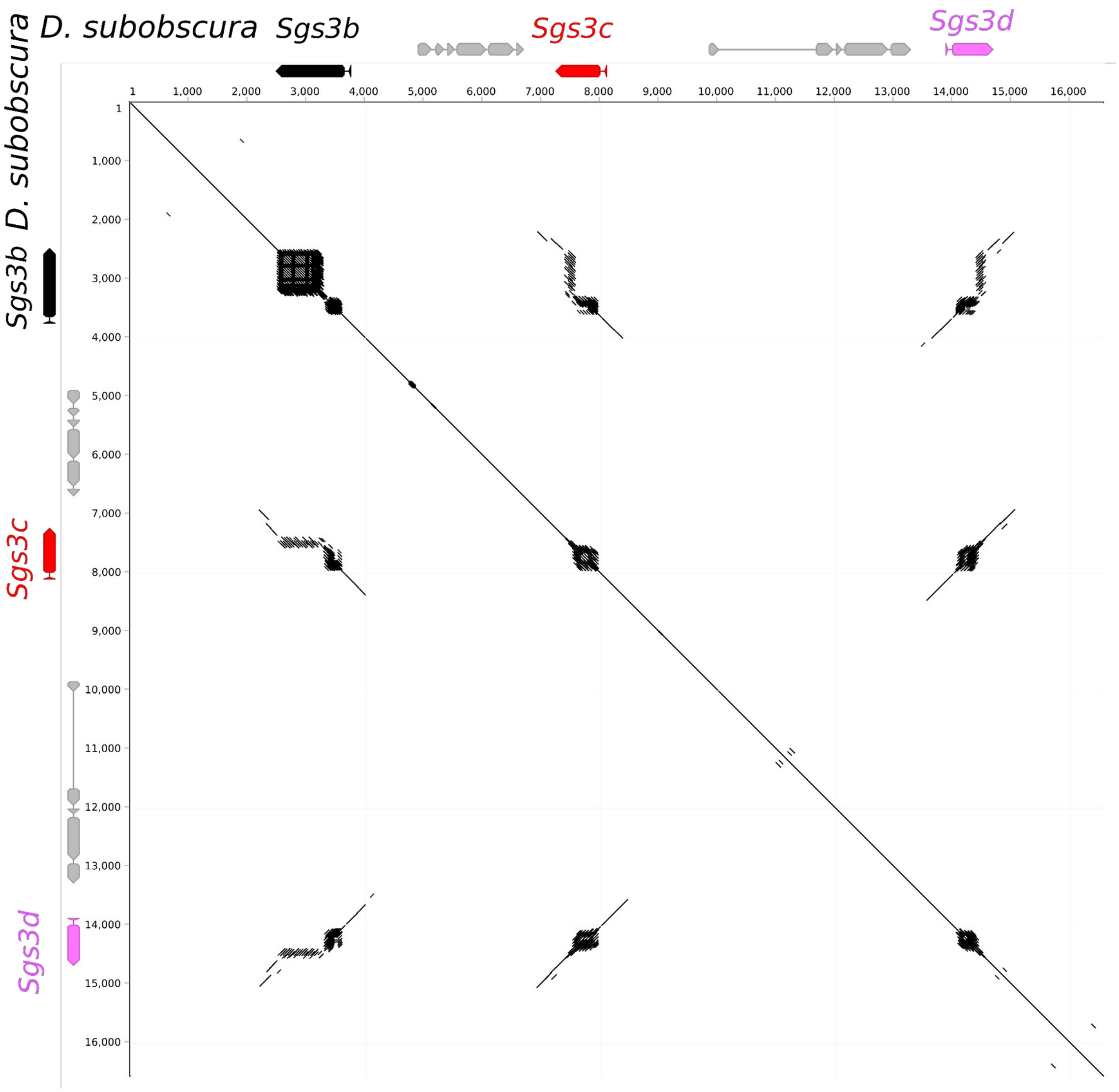

Fig S15. Dot plot comparison of *Sgs3b-d* genomic region from *D. subobscura* with itself. Same legend as Fig. S5. Black, red and light pink arrows represent *Sgs3b*, *Sgs3c* and *Sgs3d*, respectively. There are no ng genes in this region.

Supplementary Tables

Table S1. List of species and genome assemblies used in this study. All genome assemblies are PacBio-based or Nanopore-based, except the *D. eugracilis* and *D. takahashii* genome assemblies which relied on Illumina GAIIx data only. P: genome assembly based on PacBio and Illumina reads, N: genome assembly based on Nanopore and Illumina reads, I: genome assembly based on Illumina reads only.

Species	Strain	Genome Assembly	Gene annotations
D. melanogaster	iso-1	Release 6.32[P]<br>The FlyBase Consortium/Berkeley Drosophila Genome Project/Celera Genomics<br>https://www.ncbi.nlm.nih.gov/assembly/GCF_000001215.4/<br>GCA_000001215.4<br>P	FlyBase Release 6.32
D. simulans	w501	Princeton University<br>https://www.ncbi.nlm.nih.gov/assembly/GCF_016746395.1<br>GCA_016746395.1<br>P	FlyBase Release 2.01
D. sechellia	sech25	University of California, Irvine<br>https://www.ncbi.nlm.nih.gov/assembly/GCF_004382195.1<br>GCA_004382195.1<br>P	NCBI Release 101
D. mauritiania	mau12	University of California, Irvine<br>https://www.ncbi.nlm.nih.gov/assembly/GCF_004382145.1/<br>GCA_004382145.1<br>P	NCBI Release 100
D. santomea	CAGO	Princeton University<br>https://www.ncbi.nlm.nih.gov/assembly/GCF_016746245.2<br>GCA_016746245.2<br>P	This study. *Sgs* gene annotations from Da Lage et al., 2019.
D. teissieri	GT53w	Princeton University<br>https://www.ncbi.nlm.nih.gov/assembly/GCF_016746235.2<br>GCA_016746235.2<br>P	This study.
D. yakuba	NY73PB	Princeton University<br>https://www.ncbi.nlm.nih.gov/assembly/GCA_016746335.2<br>GCA_016746335.2<br>P	FlyBase Release 1.04
D. erecta	14021-0224.00,06,07	University of Arizona/ University of Chicago/ Cornell University<br>https://www.ncbi.nlm.nih.gov/assembly/GCF_003286155.1<br>GCA_003286155.2<br>P	NCBI Release 101
D. eugracilis	14026-	The modENCODE Project	NCBI Release 101

	0451.10	https://www.ncbi.nlm.nih.gov/assembly/GCF_000236325.1<br>GCA_000236325.2<br>I	
D. takahashii	14022-0311.13	Baylor College of Medicine<br>https://www.ncbi.nlm.nih.gov/assembly/GCF_000224235.1<br>GCA_000224235.2<br>I	NCBI Release 101
D. suzukii	WT3-2	Institut de Biologie du Developpement de Marseille<br>https://www.ncbi.nlm.nih.gov/assembly/GCF_013340165.1<br>GCA_013340165.1<br>P	This study. *Sgs* gene annotations from Da Lage et al., 2019.
D. biarmipes	DSSC<br>14023-0361.11	University of Pennsylvania<br>https://www.ncbi.nlm.nih.gov/assembly/GCA_005234255.1#/st<br>GCA_005234255.1<br>P	NCBI Release 101
D. ananassae	14024-0371.16-18	University of Arizona/ University of Chiago/ Cornell University<br>https://www.ncbi.nlm.nih.gov/assembly/GCF_003285975.2<br>GCA_003285975.3<br>P	NCBI Release 101
D. pseudoobscura	MV2-25<br>14011-0121.94	University of California, Irvine<br>https://www.ncbi.nlm.nih.gov/assembly/GCF_009870125.1<br>GCA_009870125.2<br>P	NCBI Release 104
D. obscura	BZ-5 IFL	Stanford University<br>https://www.ncbi.nlm.nih.gov/assembly/GCF_018151105.1<br>GCA_018151105.1<br>N	This study
D. subobscura	Ksnacht	Stanford University<br>https://www.ncbi.nlm.nih.gov/assembly/GCA_018903505.1<br>GCA_018903505.1<br>N	This study
D. rhopaloa	14029-0021.01	Stanford University<br>https://www.ncbi.nlm.nih.gov/assembly/GCF_018152115.1<br>GCA_018152115.1<br>N	This study
D. elegans	14027-0461.03	Stanford University<br>https://www.ncbi.nlm.nih.gov/assembly/GCF_018152505.1<br>GCA_018152505.1<br>N	This study
D. jambulina	14028-0671.01	Stanford University<br>https://www.ncbi.nlm.nih.gov/assembly/GCA_018152175.1<br>GCA_018152175.1<br>N	This study

D. rufa	EH091 iso-CL_3	Stanford University<br>https://www.ncbi.nlm.nih.gov/assembly/GCA_018153105.1<br>GCA_018153105.1<br>N	This study
D. kikkawai	14028-0561.14	Stanford University<br>https://www.ncbi.nlm.nih.gov/assembly/GCF_018152535.1<br>GCA_018152535.1<br>N	This study
D. triauraria	14028-0691.9	Stanford University<br>https://www.ncbi.nlm.nih.gov/assembly/GCA_018151095.1<br>GCA_018151095.1<br>N	This study
D. bipectinata	14024-0381.04	Stanford University<br>https://www.ncbi.nlm.nih.gov/assembly/GCF_018153845.1<br>GCA_018153845.1<br>N	This study
D. ficusphila	14025-0441.05	Stanford University<br>https://www.ncbi.nlm.nih.gov/assembly/GCF_018152265.1<br>GCA_018152265.1<br>N	This study

Table S2: (Table_S2.csv): Genomic coordinates of all the *Sgs* genes studied here in 24 Drosophila species.

Table S3: (.csv files compressed in a zip filev): Correspondence between NCBI gene names and the gene names used in this study, together with a description of the changes in gene annotations that have been made. ‘no change’ indicates that no modification was done on the annotations obtained from NCBI, ‘based on Borne et al, 2021 annotation’ means that the annotation was obtained from Borne et al. 2021 study. ‘annotations transferred from’ means that the gene annotation was done manually based on the existing annotation of the corresponding gene in a closely related species. There are four .csv files: (1) *Sgs1* and neighboring genes, (2) Sgs3x and neighboring genes, (3) *Sgs3/7/8* and neighboring genes, (4) *ng* genes annotated in 3C11-12, 87A1, 88C3-4 loci. In the third .csv file, ‘Newly annotated *ng* genes’ column indicates whether an *ng* gene was newly annotated in this study (‘Y’), already annotated (‘N’), or is not an *ng* (‘Not applicable’).

Table S4: (Table_S4.csv): *Sgs* exons and intron sizes for studied species. For each species, the size of the first coding exon (CDS1), intron and second coding exon (CDS2) are given in base pairs (bp). The amino acid encoded at the position of the unique phase 1 intron is also indicated.

Supplementary Files

File S1. Compressed zip file of the gene annotations (GenBank .gb files, inputs for Easyfig) of large genomic regions containing all the *Sgs* genes and their neighboring genes in the 24 studied species.

File S2. Fasta file of all the Sgs amino acid sequences used to create Figure 1B and Figure S1.

File S3. Compressed zip file of reference and corrected nucleotide sequences used to create Figure S2.

File S4. Compressed zip file of Sgs protein alignments (fasta.files) used to compute phylogenetic trees and make Weblogo figures.

File S5. *Sgs* coding sequence length in bp for species having an Sgs3x copy (.csv file, input for R script sgs_size.R).

File S6. *Sgs* coding sequence length in bp for species not having an Sgs3x copy (.csv file, input for R script sgs_size.R).

File S7. Compressed zip file of comparisons between pairs of large genomic regions (.out files obtained as outputs from Easyfig).

File S8. Table of pairwise percentage of identity between several Sgs1 and Sgs3 amino-acid sequences (.csv).

File S9. Compressed zip file of the repeats annotations (.csv files) obtained with FindRepeat in Geneious on large genomic regions for *D. melanogaster Sgs1, Sgs3/7/8, Sgs3x, D. teissieri Sgs3/7/8, D. subobscura Sgs3, D. eugracilis Sgs3.*

File S10. Compressed zip file of new glue protein alignments (.fasta files) used to make Fig. S9.

File S11. Fasta file of all the *Sgs* nucleotide sequences studied here.

File S12. Fasta file of the 154 *ng* nucleotide sequences found at loci 68C11 and 68C13.

File S13. Fasta file of the 41 *ng* nucleotide sequences found at loci 3C11-12, 28E6-28E7, 87A1 and 88C3-4.

File S14. Compressed zip file of all the R scripts (.R files) used to create the figures.

File S15. Bam file of raw reads mapped to D. rhopaloa *Sgs1* corrected nucleotide sequence, used to create Figure S2A.

File S16. Bam file of raw reads mapped to *D. ficusphila Sgs1* reference nucleotide sequence, used to create Figure S2B.

File S17. Bam file of raw reads mapped to *D. biarmipes Sgs3x* corrected nucleotide sequence, used to create Figure S2C.
