# Supplementary material for: Higher evolutionary dynamics of gene copy number for Drosophila glue genes located near short repeat sequences
Source: BMC Ecol Evol. 2024 Feb 2;24:18. doi: 10.1186/s12862-023-02178-y (PMC10835880; doi:10.1186/s12862-023-02178-y)
Supplement: Supplementary file 1 — Additional file 1. [file 12862_2023_2178_MOESM1_ESM.zip › Supplementary/list_supp_files.pdf]

Supplementary Files

File S1. Compressed zip file of the gene annotations (GenBank .gb files, inputs for Easyfig) of large genomic regions containing all the *Sgs* genes and their neighboring genes in the 24 studied species.

File S2. Fasta file of all the Sgs amino acid sequences used to create Figure 1B and Figure S1.

File S3. Compressed zip file of reference and corrected nucleotide sequences used to create Figure S2.

File S4. Compressed zip file of Sgs protein alignments (fasta.files) used to compute phylogenetic trees and make Weblogo figures.

File S5. *Sgs* coding sequence length in bp for species having an Sgs3x copy (.csv file, input for R script sgs_size.R).

File S6. *Sgs* coding sequence length in bp for species not having an Sgs3x copy (.csv file, input for R script sgs_size.R).

File S7. Compressed zip file of comparisons between pairs of large genomic regions (.out files obtained as outputs from Easyfig).

File S8. Table of pairwise percentage of identity between several Sgs1 and Sgs3 amino-acid sequences (.csv).

File S9. Compressed zip file of the repeats annotations (.csv files) obtained with FindRepeat in Geneious on large genomic regions for *D. melanogaster Sgs1, Sgs3/7/8, Sgs3x, D. teissieri Sgs3/7/8, D. subobscura Sgs3, D. eugracilis Sgs3.*

File S10. Compressed zip file of new glue protein alignments (.fasta files) used to make Fig. S9.

File S11. Fasta file of all the *Sgs* nucleotide sequences studied here.

File S12. Fasta file of the 154 *ng* nucleotide sequences found at loci 68C11 and 68C13.

File S13. Fasta file of the 41 *ng* nucleotide sequences found at loci 3C11-12, 28E6-28E7, 87A1 and 88C3-4.

File S14. Compressed zip file of all the R scripts (.R files) used to create the figures.

File S15. Bam file of raw reads mapped to D. rhopaloa *Sgs1* corrected nucleotide sequence, used to create Figure S2A.

File S16. Bam file of raw reads mapped to *D. ficusphila Sgs1* reference nucleotide sequence, used to create Figure S2B.

File S17. Bam file of raw reads mapped to *D. biarmipes Sgs3x* corrected nucleotide sequence, used to create Figure S2C.
